# Changes in m^6^A RNA methylation are associated with male sterility in wolfberry

**DOI:** 10.1186/s12870-023-04458-7

**Published:** 2023-09-29

**Authors:** Jiawen Zhao, Chujun Zhang, Sifan Li, Mengmeng Yuan, Wenlan Mu, Jing Yang, Yutong Ma, Cuiping Guan, Chuang Ma

**Affiliations:** 1 State Key Laboratory of Crop Stress Biology for Arid Areas, Center of Bioinformatics, College of Life Sciences, Northwest A&F University, Yangling, Shaanxi 712100 China; 2 College of Life Science, Ningxia University, Yinchuan, Ningxia 750021 China

**Keywords:** Differential methylation, m^6^A regulators, Male sterility, *N*^6^-methyladenosine, Wolfberry

## Abstract

**Background:**

*N*^6^-methyladenosine (m^6^A) modification is the most abundant type of RNA modification in eukaryotic cells, playing pivotal roles in multiple plant growth and development processes. Yet the potential role of m^6^A in conferring the trait of male sterility in plants remains unknown.

**Results:**

In this study, we performed RNA-sequencing (RNA-Seq) and m^6^A-sequencing (m^6^A-Seq) of RNAs obtained from the anther tissue of two wolfberry lines: ‘Ningqi No.1’ (*LB*1) and its natural male sterile mutant ‘Ningqi No.5’ (*LB*5). Based on the newly assembled transcriptome, we established transcriptome-wide m^6^A maps for *LB*1 and *LB*5 at the single nucleus pollen stage. We found that the gene *XLOC_021201,* a homolog of m^6^A eraser-related gene *ALKBH10* in *Arabidopsis thaliana*, was significantly differentially expressed between *LB*1 and *LB*5. We also identified 1642 and 563 m^6^A-modified genes with hypermethylated and hypomethylated patterns, respectively, in *LB*1 compared with *LB*5. We found the hypermethylated genes significantly enriched in biological processes related to energy metabolism and lipid metabolism, while hypomethylation genes were mainly linked to cell cycle process, gametophyte development, and reproductive process. Among these 2205 differentially m^6^A methylated genes, 13.74% (303 of 2205) were differentially expressed in *LB*1 vis-à-vis *LB*5.

**Conclusions:**

This study constructs the first m^6^A transcriptome map of wolfberry and establishes an association between m^6^A and the trait of male sterility in wolfberry.

**Supplementary Information:**

The online version contains supplementary material available at 10.1186/s12870-023-04458-7.

## Backgrounds

*N*^6^-methyladenosine (m^6^A) is one of the most prevalent chemical modifications found in eukaryotic mRNAs. With recent advances in high-throughput technologies, transcriptome-wide m^6^A modifications have been profiled for *A. thaliana* and several other plant species [1–4]. These pioneering studies demonstrated that m^6^A modification plays important roles in multiple plant growth and development processes and is responsive to a variety of biotic and abiotic stresses [5–10]. Male sterility, which is an important agronomic trait for heterosis utilization and hybrid seed production, has been reported in several plants including maize [11], wheat [12], and tomato [13]. Its underlying molecular mechanisms have been long-time investigated, resulting in the identification of tens of male sterility-related genes in *Arabidopsis thaliana* and rice [11]. These male sterility-related genes are involved in multiple biological processes including transcription and lipid metabolism [14]. Recently, RNA m^6^A modification was also found to be associated with male infertility in human studies [15]. Despite that monumental progress, the potential association of m^6^A modifications in the trait of male sterility in plants is still unknown.

*Lycium barbarum* (Goji or wolfberry) is a famous plant with medicinal and commercial value that belongs to the Solanaceae family. In China, ‘Ningqi No.1’ (denoted as *LB*1) is one of the major cultivars with multiple excellent traits including high yield, large fruit size and disease resistance. ‘Ningqi No.5’ (*LB*5) is a natural male sterile mutant of *LB*1. In contrast to *LB*1, *LB*5 plants usually exhibit abortive phenotypes such as anther shrinkage, disintegration, and vacuolization, resulting in nonfunctional anthers (i.e., no pollen in the anther) [16, 17]. Hence, *LB*1 and *LB*5 are recognized as useful materials for studying pollen development, cytoplasmic and nuclear interactions, and male fertility-related gene regulations in plants [17, 18]. However, research using this important economic crop (wolfberry) to study the trait of male sterility in plants is still scarce.

Here, we performed RNA-sequencing (RNA-Seq) and m^6^A-sequencing (m^6^A-Seq) of the anther samples of *LB*1 and *LB*5, and assembled a consensus transcriptome to derive transcriptome-wide m^6^A maps of these two wolfberry lines. By comparing the omics data generated from *LB*1 vis-à-vis *LB*5, we identified those genes with differential expression and differential m^6^A methylation patterns, and then carried out their gene ontology (GO) enrichment analysis to uncover the biological processes that may be associated with the male sterility trait in wolfberry. Our research findings provide new insight into how m^6^A can regulate the trait of male sterility in plants.

## Results

### Reference-guided assembly and annotation of the wolfberry transcriptome

**Fig. 1:**
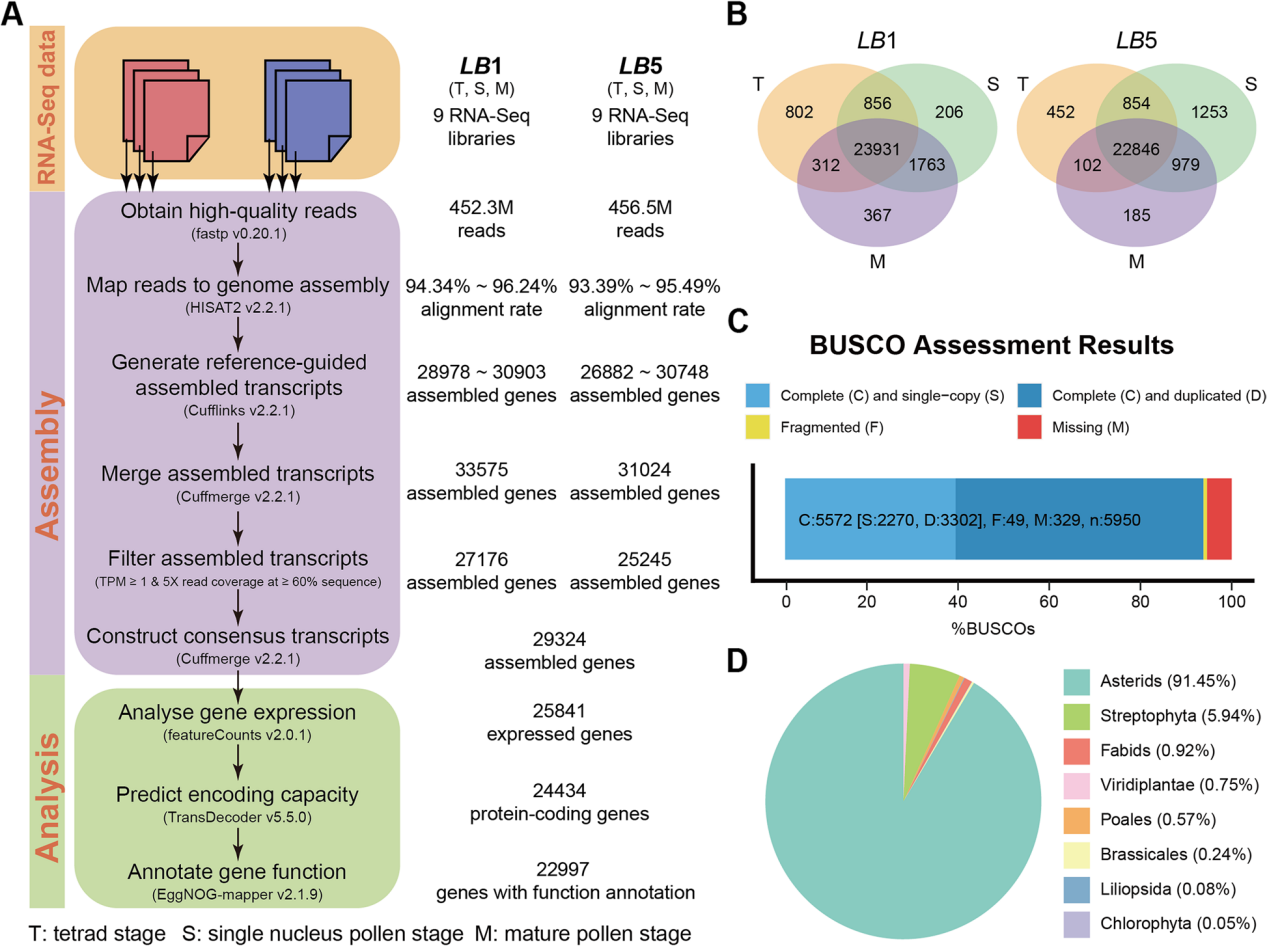
Transcriptome assembly and annotation of the two wolfberry lines. **A** Overview of wolfberry transcriptome assembly pipeline. **B** Venn diagram of genes expressed in *LB*1 and *LB*5 at three developmental stages. **C** BUSCO’s assessment results. **D** Distribution of sources for functional annotation of assembled transcripts in wolfberry

For both *LB*1 and its natural male sterile mutant *LB*5 (Figure S1), we respectively sequenced the transcriptomes of six anther samples from the tetrad stage (T), the single nucleus pollen stage (S), and the mature pollen stage (M) (*LB*1_T, *LB*1_S, *LB*1_M, *LB*5_T, *LB*5_S, *LB*5_M; see Methods), each with three biological replicates. Raw reads from each RNA-Seq library were pre-processed using fastp (v0.20.1) [19] to remove the adapter and low-quality sequences, resulting in a total of 908.8 million paired-end reads (63.5 Gb) (Table 1). After that pre-processing, the remaining reads were mapped onto the wolfberry reference genome sequences (accession number PRJNA640228) [20], using the HISAT2 program (v2.2.1) [21], whose alignment rate ranged from 93.39% to 96.24%. Then, using Cufflinks software (v2.2.1) [22], transcript fragments were first assembled for each RNA-Seq library, and then merged to generate a transcriptome assembly for both wolfberry lines under study. Under the criteria defined in a previous study [23], 29 324 genes and 84 709 transcript fragments were deemed high quality, that is, having a TPM ≥ 1 and read count ≥ 5 in at least one sample (Fig. 1A). Of these 29 324 genes, we found 25 841 that were expressed (i.e., TPM ≥ 1) in both *LB*1 and *LB*5 lines, with 2395 and 829 genes specifically expressed in each, respectively. In *LB1*, the number of specifically expressed genes decreased in the order of T stage (802) > M stage (367) > S stage (206), while in *LB5*, they were more abundant at the S stage (1253) than either the other two stages (T: 452 and M: 185) (Fig. 1B; Table S1).

**Table 1:** Comparison of library size, library quality, and read alignment rates of RNA-Seq samples

**Libraries**	**Total reads**	**% > Q30**	**Q30 bases (Mb)**	**GC content %**	**Alignment rate %**
*LB*1_T_1	43719178	95.2	6181.9	42.5	95.3
*LB*1_T_2	55707414	94.2	7785.9	42.4	94.9
*LB*1_T_3	54023534	94.6	7581.0	42.5	94.9
*LB*1_S_1	42051874	95.1	5936.2	42.6	95.0
*LB*1_S_2	49105268	94.9	6912.6	42.7	95.0
*LB*1_S_3	54664072	94.2	7633.6	42.6	94.3
*LB*1_M_1	52333548	94.9	7372.8	42.6	95.4
*LB*1_M_2	48417410	94.4	6776.3	42.6	94.8
*LB*1_M_3	52375878	96.5	7530.3	42.6	96.2
*LB*5_T_1	40563132	97.0	5863.5	42.7	95.4
*LB*5_T_2	40153692	96.3	5759.2	42.5	95.1
*LB*5_T_3	53393094	96.9	7711.8	42.8	95.3
*LB*5_S_1	54215340	94.8	7627.5	42.5	94.5
*LB*5_S_2	54258376	94.8	7629.5	42.6	93.9
*LB*5_S_3	58491290	94.4	8186.0	42.3	93.4
*LB*5_M_1	59103888	94.9	8319.5	42.6	94.1
*LB*5_M_2	40186828	96.9	5807.5	42.5	95.5
*LB*5_M_3	56066746	95.1	7911.9	42.3	94.1

Q30 demonstrates the sequencing error rate < 0.1%

Overall, 83.3% (24 434) of 29 324 annotated genes (Table S2) were predicted capable of encoding proteins by TransDecoder [24]. These 24 434 protein-encoding genes (PCGs) served as the input of BUSCO [25] to evaluate the completeness of the assembled transcripts. BUSCO’s assessment results revealed that of the 5950 core genes queried, 94.47% were detected (5574 complete and 49 partial) (Fig. 1C). Among these 24 434 PCGs, we found that 22 997 (94.12%) can be assigned with a function annotation label when using EggNOG-mapper (emapper) [26]. The vast majority of (90%) this annotation information came from the clade of asterids (e.g., *Solanum lycopersicum*, *Solanum tuberosum*) that are closely related taxonomically to wolfberry (Table S3; Fig. 1D). In addition, 1517 of these 24 434 PCGs (Table S4) were predicted to be transcript factors (TFs) according to PlantTFDB v5.0 [27].

Overall, the assembled transcriptome, along with its corresponding functional annotations, provided a comprehensive resource now available for the transcriptome-wide m^6^A analysis and other functional genomics studies in wolfberry.

### Identification and expression analysis of m^6^A potential regulators in wolfberry

**Fig. 2:**
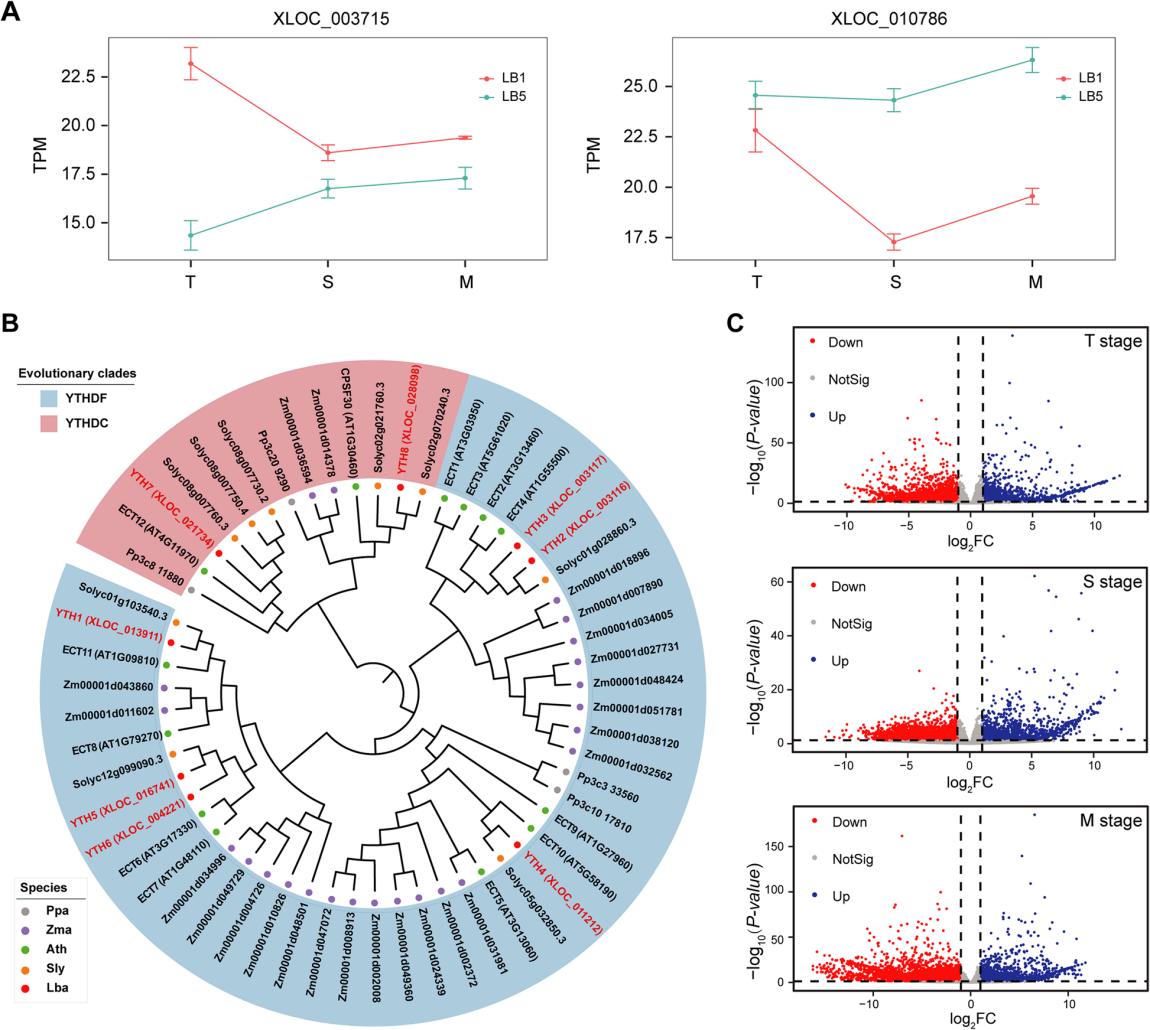
Identification and expression analysis of putative m^6^A regulators in wolfberry. **A** Dynamic trends in the expression levels of two example genes. **B** Phylogenetic tree of the YTH gene family (Ppa: *Physcomitrella patens*, Zma: *Zea mays*, Ath: *Arabidopsis thaliana*, Sly: *Solanum lycopersicum* and Lba: *Lycium barbarum*); the color-marked genes are annotated YTH genes in wolfberry. **C** Volcano plot of differentially expressed genes at each stage (*p*-value < 0.05, absolute fold-change > 2)

A total of 25 m^6^A potential regulators (7 writers, 10 erasers, and 8 readers) in wolfberry were identified from wolfberry’s assembled transcriptome by applying a specified bioinformatics pipeline (Fig. 2B; Table 2; Figures S2 and S3). These potential regulators were named according to their predicted orthologs from *Arabidopsis* [28] and tomato [29]. Among these 25 potential regulators, 22 were relatively highly expressed with TPM ≥ 10 in both *LB*1 and *LB*5 lines. These 22 genes exhibited dynamic expression patterns across T to M stages in either line (Figure S4). Several genes had markedly divergent expression dynamics between *LB*1 and *LB*5. For example, certain m^6^A writer genes, namely *XLOC_003715* (*LbaMTC*, homologous with *AtMTC*) and *XLOC_10786* (*LbaMTB*, homologous with *AtMTB*; Figure S3B), displayed different expression patterns when going from the T to M stages between *LB*1 and *LB*5 (Fig. 2A). There were 14 genes whose expression was greater in *LB*5 than *LB*1 at all three stages studied. One representative example is *XLOC_016741* (*LbaYTH5*) encoding an YTH domain-containing protein. Gene *LbaYTH5* is close to the experimentally demonstrated *Arabidopsis* m^6^A readers *AtECT6* and *AtECT7* in the phylogenetic tree, constructed with the FastTree software [30] using YTH domain-containing proteins from wolfberry, tomato, maize, *A. thaliana* and *Physcomitrium patens* (Fig. 2B). In *LB*1, the expression level of *LbaYTH5* was 68.64, 51.97, and 56.27 at the T, S, and M stages, but higher at 76.26, 83.20, and 104.35 in *LB*5, respectively (Figure S5).

**Table 2:** List of putative m^6^A regulators in wolfberry

**Gene ID**	**m**^**6**^**A regulator**	**Annotation of *Arabidopsis***	**Nomenclature name**
XLOC_028357	eraser	ALKBH9	*ALKBH9-like*
XLOC_027737	eraser	ALKBH9	*ALKBH9-like*
XLOC_013931	eraser	ALKBH9	*ALKBH9-like*
XLOC_022826	eraser	ALKBH10	*ALKBH10-like*
XLOC_021201	eraser	ALKBH10	*ALKBH10-like*
XLOC_001156	eraser	ALKBH10	*ALKBH10-like*
XLOC_023876	eraser	ALKBH2	*ALKBH2-like*
XLOC_016642	eraser	ALKBH8	*ALKBH8-like*
XLOC_023502	eraser	ALKBH1	*ALKBH1-like*
XLOC_003094	eraser	ALKBH6	*ALKBH6-like*
XLOC_028098	reader	CPSF30	*LbaYTH8*
XLOC_021734	reader	ECT12	*LbaYTH7*
XLOC_011212	reader	ECT10	*LbaYTH4*
XLOC_004221	reader	ECT6/7	*LbaYTH6*
XLOC_016741	reader	ECT6/7	*LbaYTH5*
XLOC_013911	reader	ECT11	*LbaYTH1*
XLOC_003116	reader	ECT2/4	*LbaYTH2*
XLOC_003117	reader	ECT2/4	*LbaYTH3*
XLOC_009741	writer	MTA	*LbaMTA*
XLOC_010786	writer	MTB	*LbaMTB*
XLOC_003715	writer	MTC	*LbaMTC*
XLOC_006042	writer	VIR	*LbaVIR*
XLOC_006771	writer	HAKAI	*HAKAI-like*
XLOC_005589	writer	HAKAI	*HAKAI-like*
XLOC_007322	writer	FIP37	*LbaFIP37*

Lba represents the *Lycium barbarum*

Pair-wise comparisons also revealed that genes of some m^6^A potential regulators differed starkly in their expression between *LB*1 and *LB*5. Using DESeq2 [31], we identified 4499, 4575, and 7491 differentially expressed genes (DEGs) between *LB*1 and *LB*5 at the T, S, and M stages, respectively (Fig. 2C; Table S5). One representative gene is *XLOC_021201*, which may encode an m^6^A eraser regulator, belonged to the same evolutionary branch as the *Arabidopsis* gene *AtALKBH10* (Figure S3A)*.* Compared with *LB*5, this gene had markedly higher values of TPM in *LB*1 at both S and M stages (Figure S4). Altogether, these results revealed the differential expression patterns of genes encoding m^6^A potential regulators between *LB*1 and *LB*5 during the anther development of wolfberry from the T to M stage.

### Transcriptome-wide identification of m^6^A methylation in *LB*1 and *LB*5

**Fig. 3:**
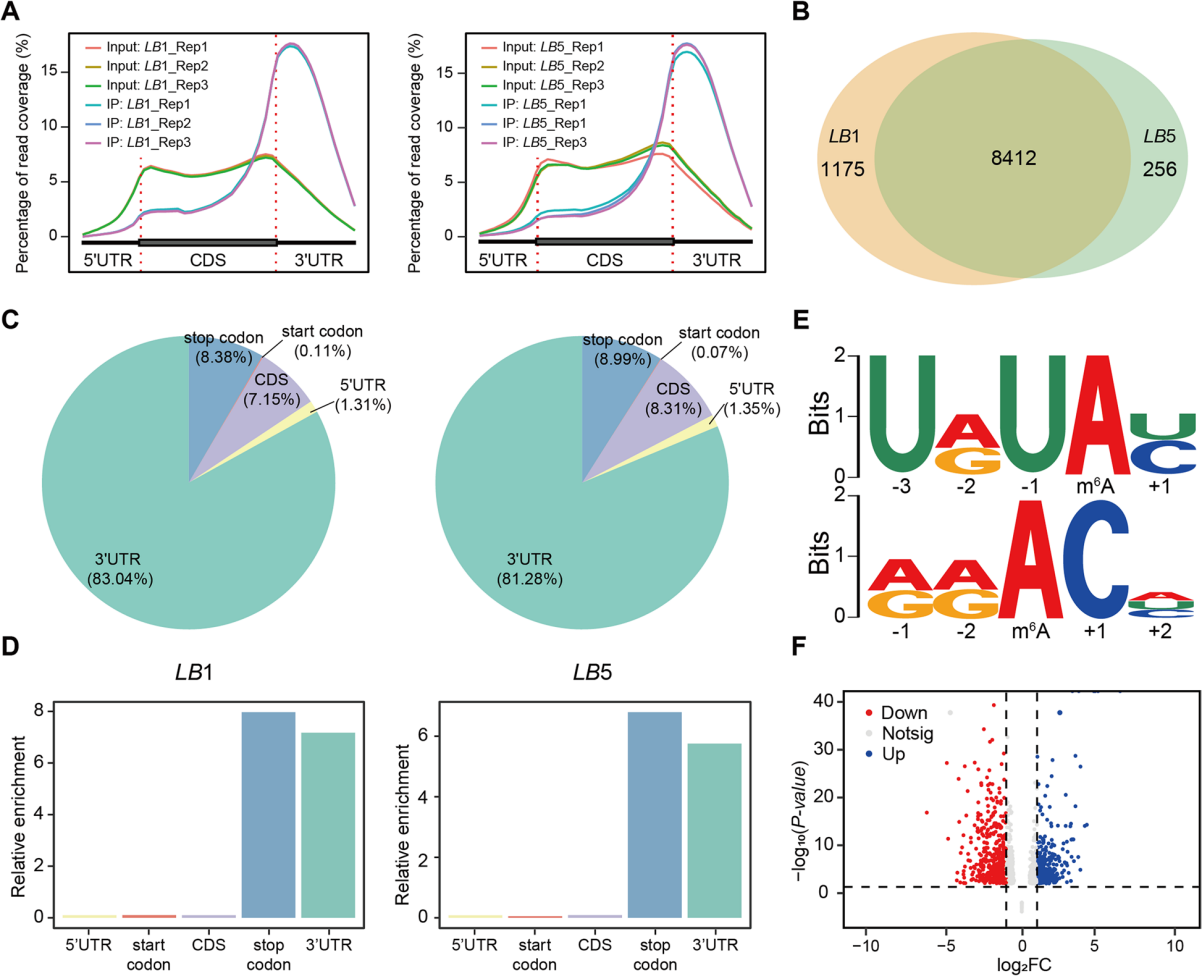
Overview of the m^6^A methylome in wolfberry. **A** The read distribution of input and IP data from m^6^A-Seq of *LB*1 and *LB*5. **B** Comparison of m^6^A peaks between *LB*1 and *LB*5. **C** Peak density in five non-overlapping transcript segments: the 5’-untranslated region (5’UTR), near start codon, coding sequence (CDS), near stop codon, and 3’-untranslated region (3’UTR). **D** Relative enrichment of the m^6^A peaks in the five non-overlapping transcript segments. **E** The motif on the top is the 1^st^-ranked enriched URUAY motif (where R denotes A/G, A is m^6^A and Y denotes C/U). The motif on the bottom is the canonical RRACH motif (where R denotes A/G, A is m^6^A, and H denotes A/C/U). **F** Volcano plot of differentially methylated genes (*p*-value < 0.05, absolute fold-change > 2)

With the DEGs encoding m^6^A potential regulators in mind, we then asked whether there some differences in the m^6^A methylome also arise between *LB*1 and *LB*5. To address this question, we firstly measured the m^6^A/A ratio of pollens from these two wolfberry lines at the S stage, and found that the m^6^A level in mRNA was slightly different between *LB*1 and *LB*5 (Figure S6). Then, we used anther samples at the S stage as an example to obtain m^6^A maps of *LB*1 and *LB*5 via m^6^A-Seq technology. Six m^6^A-immunoprecipitation (IP) and matched input (non-IP control) libraries were constructed and sequenced for RNAs from the anther samples of these two lines at the S stage, with three biological replicates per sample. Raw sequencing reads from each library were processed to discard adaptor sequences and low-quality bases using the fastp (v0.20.1) [19]. The resulting reads from the wolfberry *LB*1 and *LB*5 samples were aligned to the wolfberry reference genome (accession number PRJNA640228) using HISAT2 (v2.2.1) [20, 21]. Read distribution analysis showed that the reads from m^6^A-IP samples accumulated highly around the stop codon and within the 3’-untranslated region (3’UTR) in all samples, with the sequencing data of input and IP being highly correlated between replicates, thus confirming the high quality of m^6^A-Seq in this study (Fig. 3A).

We detected 10 389 and 9301 m^6^A peaks in *LB*1 and *LB*5, corresponding to 9587 and 8668 m^6^A-modified genes, respectively (Fig. 3B; Table S6). In both *LB*1 and *LB*5 lines, their m^6^A-modified genes (m^6^A genes) were significantly longer than genes without m^6^A peaks (non-m^6^A genes) (Student’s t-test, *p*-value < 0.001) (Figure S7). In addition, those m^6^A-modified genes exhibited greater expression than non-m^6^A genes (Figures S8 and S9). Moreover, m^6^A peaks were enriched in the following order: 3’UTR (3’-untranslated region) > near stop codon > CDS (coding sequence) > start codon > 5’UTR (5’-untranslated regions) (Fig. 3C, D). All these m^6^A peaks were scanned further for enriched motifs, using MEME suite (http://meme-suite.org/index.html) [32]. As expected, the URUAY (where R represents A/G and Y represents C/U; Fig. 3E) motif was significantly enriched within the m^6^A peaks, and in both lines the URUAY motif is the most enriched one. We next examined the canonical m^6^A motif RRACH (where R represents A/G, A is m^6^A, and H represents A/C/U; Fig. 3E), using another commonly used motif analysis program, HOMER (v4.10) [33]. Evidently, the RRACH motif could also be detected in our m^6^A-Seq data, it being significantly enriched in m^6^A peaks vis-à-vis non-m^6^A regions (Figure S10).

Furthermore, a differential methylation analysis was performed by RADAR [34], which uncovered 2205 genes that were differentially m^6^A-modified between *LB*1 and *LB*5. Among these 2205 differentially m^6^A-modified genes, 1642 genes (including 67 TFs) were hypermethylated in line *LB*1 compared with line *LB*5, while 563 genes (including 19 TFs) were hypomethylated (Table S7; Fig. 3F). Previous studies have linked a number of TFs from MADS, MYB, ARF, and GATA gene families to plant male sterility [35–39]. In our study, 86 differentially m^6^A-modified genes may have encoded members of those TF families (i.e., bHLH: 11, B3: 6, WRKY: 5, NAC: 5). Interestingly, we found that the gene *XLOC_003715* encoding an m^6^A writer also had differential methylation between the *LB*1 and *LB*5 lines (Figure S3B). Further, the gene *XLOC_027737* that may encode an m^6^A demethylase was specifically hypermethylated in *LB*1 (Figure S3A). These results suggested that m^6^A methylation at the RNA level may form a complex framework for epigenetic regulation of male sterility in wolfberry.

### Functional enrichment analysis of hypermethylated genes in wolfberry

**Fig. 4:**
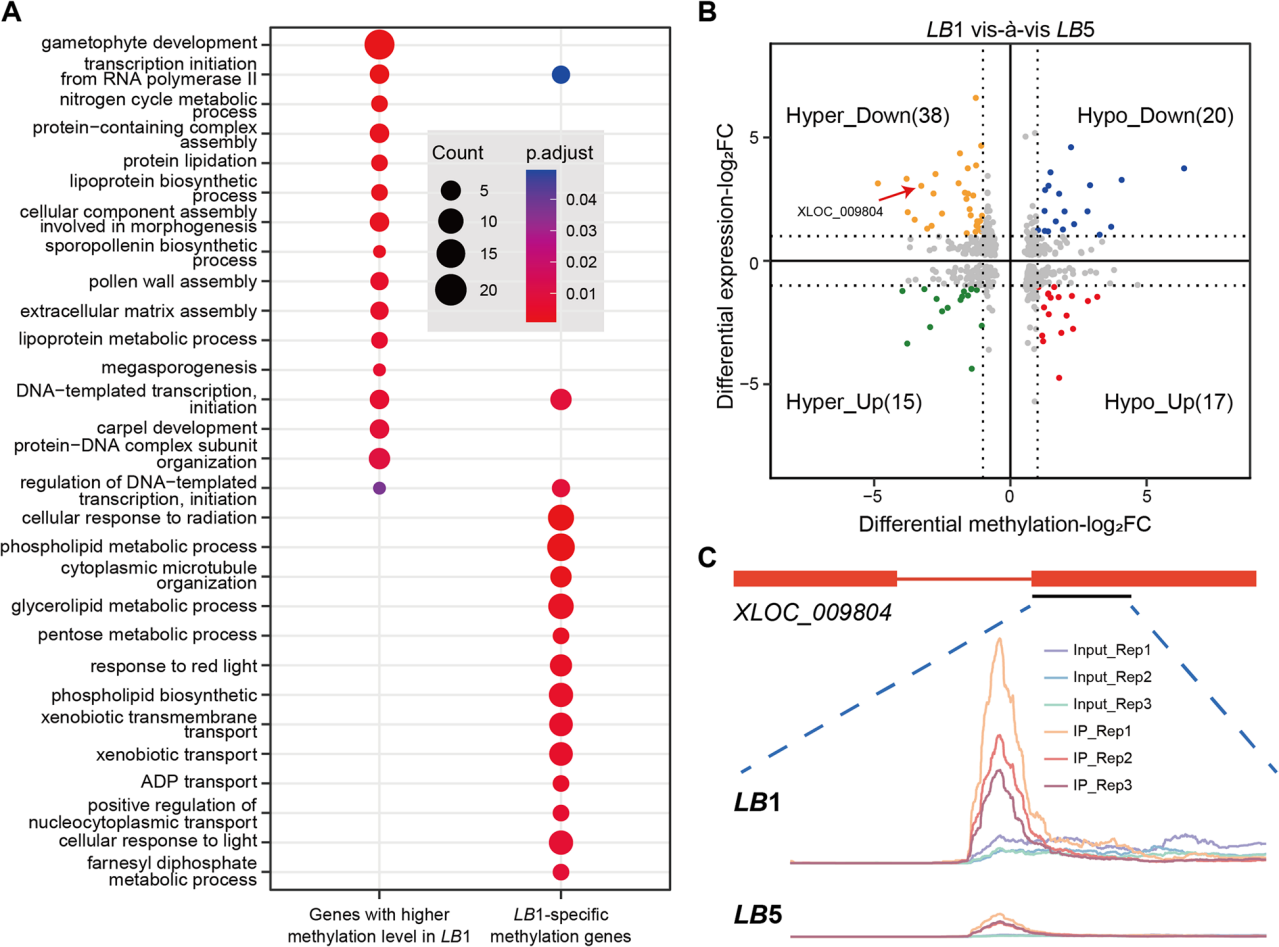
Function enrichment analysis of the hypermethylated genes. **A** Scatterplot of the GO enrichment analysis of hypermethylated genes. **B** Volcano plot of DEGs and differentially methylated genes (*p*-value < 0.05, absolute fold-change > 2). **C** Enrichment of the m^6^A methylation of gene *XLOC_009804* between *LB*1 and *LB*5

Among 1642 hypermethylated genes, 1175 were *LB*1-specific methylation genes while the other 467 genes featured higher m^6^A methylation levels in *LB*1 than *LB*5. Gene ontology (GO) enrichment analysis revealed these 1175 *LB*1-specific methylation genes being mainly enriched in biological processes, such as energy metabolism and biosynthesis, photosynthesis, lipid metabolism and biological component synthesis (Fig. 4A; Table S8). As an essential process in anther and pollen development, the destruction of lipid metabolism has been reported to lead to male sterility [14, 40]. Here, we found that 44 *LB*1-specific methylation genes, including *XLOC_002359* (GATase), *XLOC_002916* (Lipoxygenase), and *XLOC_010833* (Cytochrome p450, CYP), were annotated to the lipid metabolic process (Tables S3 and S8).

Similarly, the GO enrichment analysis showed that, for the 467 genes with higher methylation level in *LB*1 than *LB*5, they were also specifically enriched in biological processes, namely those associated with gametophyte development, pollen wall assembly and lipoprotein biosynthetic (Fig. 4A; Table S8). As other research has shown, pollen development is affected by pollen wall assembly in maize, wheat, and rice; abnormally developed pollens can cause male sterility [41–43]. We also found that 53 of these 467 genes were both differentially methylated and differentially expressed between *LB*1 and *LB*5 at the S stage (15 genes up-regulated and 38 genes down-regulated in *LB*1). One representative gene is *XLOC_009804*, encoding a bHLH TF, which showed hypermethylation and down-regulation in *LB*1 (Fig. 4B, C). In alfalfa plants, a tapetum-specific bHLH TF has been identified to play a regulatory role in the process conferring male sterility [44]. These results indicated that at least some of hypermethylated genes were closely linked to the trait of male sterility in wolfberry.

### Functional enrichment analysis of hypomethylated genes in wolfberry

**Fig. 5:**
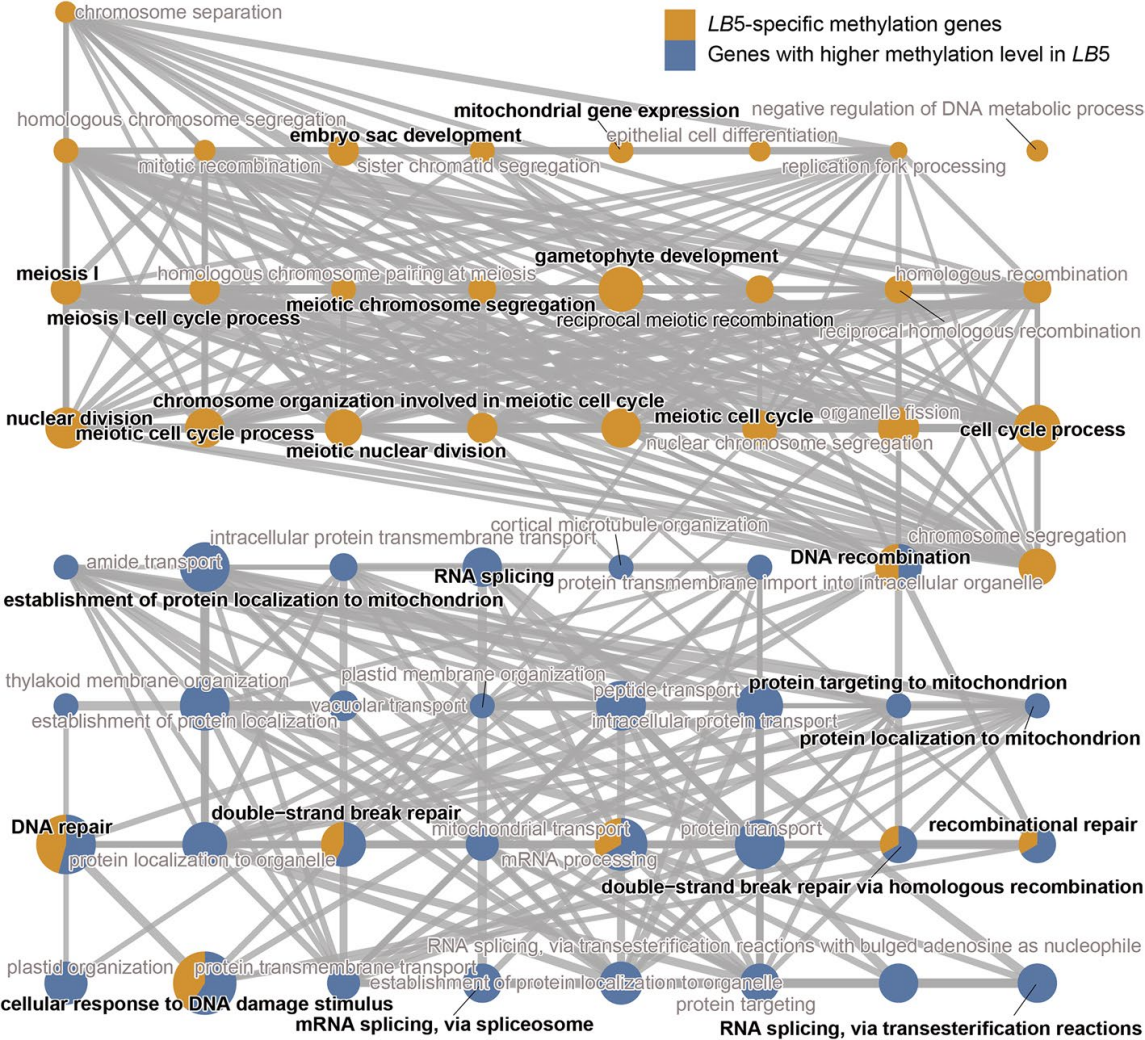
Function enrichment analysis of hypomethylated genes. Network plot of GO enrichment analysis of hypomethylated genes (yellow: *LB*5-specific methylation genes; blue: genes with higher methylation level in *LB*5). The size of the circle represents the number of genes enriched in the GO terms

We further explored whether (or not) hypomethylated genes were associated with infertility in wolfberry. Of the 563 hypomethylated genes, 256 were *LB*5-specific methylation genes while the other 307 genes underwent a higher m^6^A methylation level in *LB*5 than *LB*1. GO enrichment analysis showed that these 256 *LB*5-specific methylation genes were mainly enriched in the cell cycle process, gametophyte development, and reproductive process (Fig. 5; Table S9). Being the most critical cell type for plant fertility and crop production, gametocyte development is directly linked to the formation of plant male sterility. Many of these pathways have been implicated in gametocyte development; for example, the cell cycle and reproductive processes directly affect the two pivotal stages: microsporogenesis and microgametogenesis [45]. Further, the 256 genes with a higher methylation level in *LB*5 were also specifically enriched in biological processes, such as cellular response to DNA damage, double-strand break repair, and DNA repair (Fig. 5; Table S9). Extensive studies of rice have shown that sustained double-strand breaks cause the programmed cell death of male gametes and complete male sterility [46–49].

To gain deeper insight into the functioning of these hypomethylation genes, we performed a combinational analysis of transcriptome and m^6^A methylome in the anthers of *LB*1 and *LB*5 at the S stage. We found that 62 *LB*5-specific methylation genes are concomitantly differentially expressed (9 genes up-regulated and 53 genes down-regulated in *LB*1). One representative example is *XLOC_020363*, which encodes a TF belonging to the ERF gene family. The ERF gene family is thought to be involved in MYB regulation of pollen ablation and male sterility in canola [50]; our results suggested that at least some of hypomethylated genes may also participate in the trait of male sterility in wolfberry.

Collectively, these results suggested that m^6^A-modified genes figure prominently in how male sterility arises in wolfberry, and that gaining mechanistic insight into the functions of genes with both differential expression and differential methylome patterns is imperative for a better understanding of male sterility in plants.

## Discussion

In the present study, we performed a series of m^6^A-Seq and RNA-Seq experiments on the anther samples of two wolfberry lines *LB*1 and *LB*5, and carried out in-depth bioinformatics analysis to establish the association between m^6^A modification and the male sterility trait in wolfberry.

### Constructing the first m^6^A transcriptome map of wolfberry

Using the generated RNA-Seq datasets, we assembled a consensus transcriptome of wolfberry consisting of 29 324 genes and 84 709 transcript fragments (Fig. 1A; https://github.com/cma2015/Wolfberry_m6A). This addressed the problems caused by the lack of public availability of gene annotations for wolfberry. Based on the newly assembled transcriptome, we constructed the first m^6^A transcriptome map of wolfberry. The distribution of m^6^A within different gene contexts in wolfberry anther is similar to that reported in *Arabidopsis* [7], maize [1] and a few other under-studied plant species [2]. However, the top ranked motifs (and significance) enriched within m^6^A peaks in wolfberry anther (URUAY [Y = C/U] and RRACH [R = A/G, H = A/C/U]; Fig. 3E) differed from those reported in the anther of tomato (only URUAH) [9] as well as rice (WKUAH) [51]. This difference may represent the unique biological significance of m^6^A methylation among these species. We hope that the constructed m^6^A transcriptome map for the anther of wolfberry will be helpful to further understanding the regulatory mechanism of m^6^A in the anthers of plants [52].

### Linking m^6^A-modified genes to the trait of male sterility in wolfberry

Plant male sterility is the outcome of a complex process that has been associated with abnormalities in growth substance metabolism, epigenetic regulation, and mitochondrial function [14, 35, 53–55]. So far, however, the association of m^6^A methylation with male infertility has only been explored in humans [15]. Our results show that an m^6^A potential regulator is differentially expressed between *LB*1 and *LB*5 (Figures S3A and S5). In addition, both hypermethylated and hypomethylated genes between *LB*1 and *LB*5 are involved in multiple biological processes (e.g., transcript and lipid metabolism) (Tables S8 and S9), which have been previously reported to be related to the trait of male sterility in plants. Among these differentially m^6^A modified genes, some are homologous to those of already annotated male sterility-related genes. Figure S11 depicts two representative genes, one encoding a lipoxygenase and the other encoding a bHLH transcription factor, with high evolutionary conservation of motif constituents between wolfberry and *Arabidopsis*. These observation results suggest the association between m^6^A and the trait of male sterility in wolfberry.

**Fig. 6:**
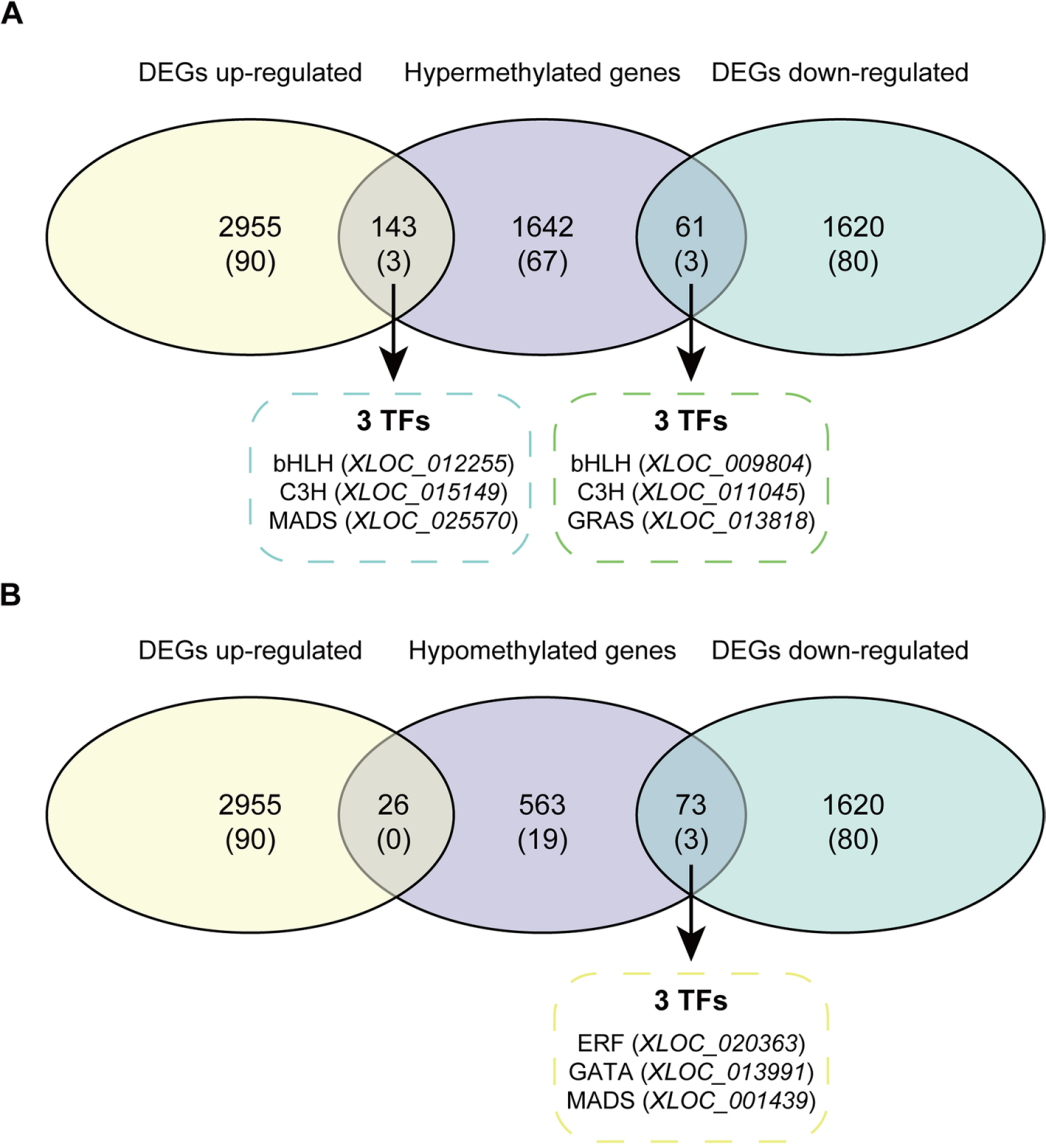
Venn diagram of numbers of differentially methylated genes and differentially expressed genes. **A** Venn diagram of numbers of hypermethylated genes and differentially expressed genes. **B** Venn diagram of numbers of hypomethylated genes and differentially expressed genes. Numbers in parentheses represent the number of TFs

**Fig. 7:**
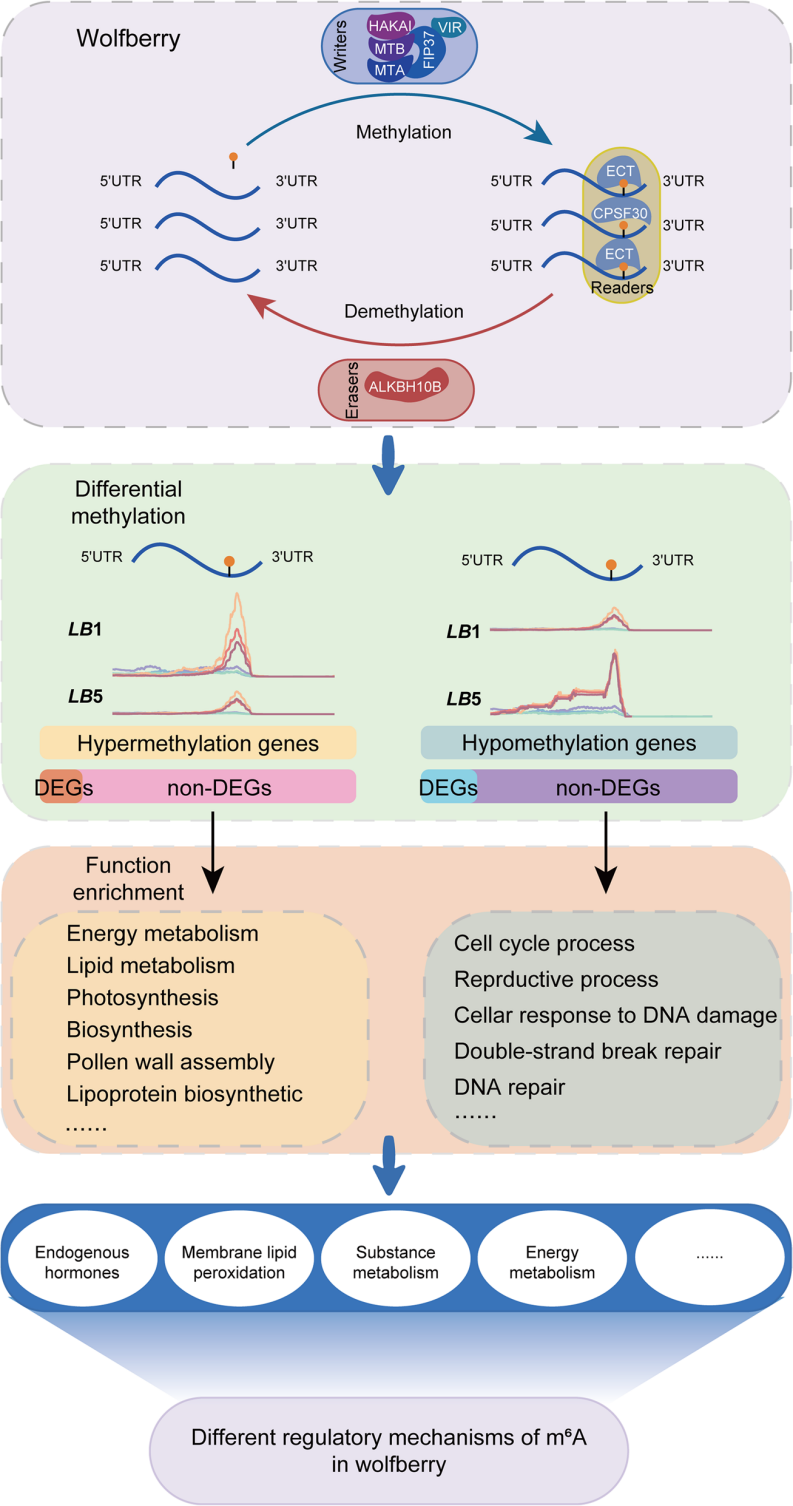
Schematic illustration of potential m^6^A regulatory mechanisms in wolfberry

The integrative analysis of the transcriptome and m^6^A methylatome also enabled us to identify 4575 DEGs and 2205 differentially m^6^A-modificated genes (1642 hypermethylated and 563 hypomethylated genes) between *LB*1 and *LB*5. We found that 303 genes (including 9 TFs) were both differentially expressed and differentially methylated (Fig. 6; Table S10). This preliminary result shows that changes to m^6^A methylation could occur in genes with and without differential expression, which suggests that m^6^A modification may have different regulatory mechanisms in wolfberry (Fig. 7).

### Limitations and future work

Despite these preliminary findings, there are still some limitations to this analysis that can be pursued as further work. Firstly, we applied the m^6^A-Seq technology to investigate the patterns of m^6^A modification peaks in the anther samples of *LB*1 and *LB*5 at the S stage. Further considering the m^6^A methylation changes across different developmental stages and using m^6^A sequencing technologies with single-nucleotide resolution will help us to improve the association between m^6^A modification and the trait of male sterility. Secondly, we only established the potential association between m^6^A modification and the trait of male sterility. More work, including ‘wet’ experiments and advanced bioinformatics analysis [56], is still needed to carefully validate whether m^6^A modification is a contributor to the male sterility trait or not. Thirdly and lastly, we computationally identified some m^6^A-modified genes of interest in wolfberry. In-depth study of the functions of these genes will contribute to field of plant genetics and breeding.

## Conclusions

We show that the m^6^A landscape is altered in *LB*1 and its natural male sterile mutant *LB*5. Hence, changes to and from m^6^A methylation may be associated with the trait of male sterility in wolfberry. The modulation of m^6^A epitranscriptome might be a promising target for breeding male sterility lines in the future improvement of wolfberry and other crops.

## Material and methods

### Plant materials

The flower buds of *LB*1 and *LB*5 were harvested from the Quanxing Wolfberry Plantation (Zhongning County, Ningxia, China) at three developmental stages: (1) the tetrad (T) stage, when the flower bud length is 3.0–5.0 mm; (2) the single nucleus pollen (S) stage, when the flower bud length is 5.0–7.0 mm; (3) the mature pollen (M) stage, when the flower bud length is greater than 8.0 mm. For each developmental stage, three biological replicates were collected, each of which contained samples from at least three plants. After their collection, the samples of anthers were rapidly stripped on the dry ice, sub-packaged into centrifuge tubes, and frozen immediately in liquid nitrogen, then stored in − 80 °C freezer until used. These frozen plant materials were preserved in the sample storage room of College of Life Science, Ningxia University, China.

### RNA isolation and library preparation for sequencing

Total RNA was isolated from the flower bud samples of *LB1* and *LB5* using the TRIzol reagent (Invitrogen, Carlsbad, CA, USA) following the manufacturer’s instructions. For each sample, the NanoDrop ND-1000 (NanoDrop, Wilmington, DE, USA) was used to determine the amount and purity of total RNA. The completeness of total RNA was assessed by using the Bioanalyzer 2100 (Agilent, CA, USA), and the electrophoresis with denaturing agarose gel was used to confirm the results.

### High-throughput RNA-Seq and m^6^A-Seq

For the RNA-Seq libraries, clustering of index-coded samples was performed on a cBot Cluster Generation System by using the TruSeq PE Cluster Kit v3-cBot-HS (Illumia) according to the manufacturer’s instructions. After cluster generation, the library preparation was sequenced on the Illumina HiSeq 2000 platform, from which the paired end reads (2 × 150 bp) were generated. For the m^6^A-Seq libraries, the m^6^A-Seq was performed as previously described [2]. Briefly, the Poly (A) RNA was purified from the 50 μg of total RNA using Dynabeads Oligo (dT)25-61005 (Thermo Fisher, CA, USA). After that, the Poly (A) RNA was truncated into small fragments using the Magnesium RNA Fragmentation Module (NEB, cat.e6150, USA). The ensuing truncated RNA small fragments were incubated with m^6^A-specific antibodies (No. 202003, Synaptic Systems, Germany) in an IP buffer (50 mM Tris–HCl, 750 mM NaCl, and 0.5% IgepalCA-630). And the RNA incubated with m^6^A-specific antibodies (IP RNA) was reverse transcribed using SuperScript™ II (Invitrogen, cat. 1896649, USA) to generate the complementary DNA (cDNA); Then, the U-labeled second-stranded DNAs was synthesized with *E.coli* DNA polymerase I (NEB, cat.m0209, USA), RNase H (NEB, cat.m0297, USA) and the dUTP Solution (Thermo Fisher, cat.R0133, USA).

Next, the A base, which connected to the indexed adapter, was added to the blunt end of each chain. The T base dangle on each adapter of each chain was used to connect the adapter to the A-tailed segment DNA. After that, both single- and dual-index adapters were connected to the fragments, and AMPureXP beads was used for size selection. After treatment of U-labeled second-strand DNAs with heat-labile UDG enzyme (NEB, cat.m0280, USA), the ligation products were amplified by PCR under conditions consistent with the previously described [2]. The mean insert size for the final cDNA library was 300 bp with SD 50. Finally, the 2 × 150 bp paired-end sequencing (PE150) was performed on an illumina Novaseq™ 6000 (LC-Bio Technology CO., Ltd., Hangzhou, China), this done following the vendor’s recommended protocol.

### Reference-guided transcriptome assembly for wolfberry

In this study, the reference genome of *L. barbarum* (wolfberry) was downloaded from the GenBank (https://www.ncbi.nlm.nih.gov/genbank) repository at the National Center for Biotechnology Information (NCBI) [57] under accession number PRJNA640228 [20]. To perform the reference-guided transcriptome assembly, the quality of RNA-Seq data was first examined using MultiQC (v1.13.dev0) [58], after which any adapter and low-quality reads were removed using fastp (v0.20.1) [19]. Once the quality control was completed, the remaining clean reads were mapped to the genome sequences using the fast, splice-aware alignment program HISAT2 (v2.2.1) [21], this yielding the BAM (binary sequence alignment format) file recording read-genome alignments. Cufflinks (v2.2.1) software [22] was used to perform the transcriptome assembly. Finally, the RNA-Seq assembly results from the same line were merged using the *Cuffmerge* tool in Cufflinks (v2.2.1).

### Construction of the consensus transcriptome for the two wolfberry lines

We screened the assembled transcript fragments on the basis of expression-level evidence [23]. The TPM (Transcripts Per Million) values were calculated using featureCounts (v2.0.1) [59] and read counts per base were calculated using bedtools *genomecov* (v2.30.0) [60]. Those assembled transcripts with either low expression (e.g., TPM < 1) or low read coverage (e.g., < 5) in the majority of each transcript fragments (e.g., 60%) were discarded. Finally, we merged the remaining high-confidence transcript fragments from two lines to construct a consensus transcriptome, and further calculated the TPM for the latter using featureCounts (v2.0.1) [59].

### Gene expression analysis

To investigate the conditions under which certain genes are specifically expressed, their expression levels (TPM values) in samples from different developmental stages were compared. For each line, a gene was considered as expressed under a condition if it was expressed (e.g., TPM ≥ 1) in at least one of the three biological replicates. The R package ‘DESeq2’ [31] was used to search for DEGs (differentially expressed genes) based on the criteria of *p*-value < 0.05 and absolute fold-change > 2.

### Gene structure and functional annotation

To predict the open reading frame (ORF) of assembled transcript fragments, we used the transcripts coding regions-finding software TransDecoder (https://github.com/TransDecoder/TransDecoder/releases, v5.5.0) [61]. The fast functional annotation tool EggNOG-mapper (emapper v2.1.9; eggNOG DB v5.0.2, http://eggnog5.embl.de; diamond vv2.0.1) [22, 62, 63] was used to annotate the assembled transcript fragments under these parameters: tax_scope = ‘Viridiplantae’. Further, the transcription factor information was simultaneously annotated using the Plant Transcription Factor Database (PlantTFDB v5.0, http://plantregmap.gao-lab.org) [27].

### Annotation of putative m^6^A regulators in wolfberry

Six HMM (hidden Markov model) profiles of the m^6^A regulators were downloaded from the Pfam database (http://pfam-legacy.xfam.org) [64], namely eraser: ALKBH10B (PF13532), reader: YTH (PF04146), and writer: MT-A70 (PF05063), FIP37 (PF17098), VIR (PF15912), HAKAI (PF18408). These HMM profiles were respectively inputted into the *hmmsearch* program of HMMER software (v3.1b2) [65] to search the HMM-profile domain against known wolfberry protein sequences, following by building a new HMM profile for wolfberry, using the *hmmbuild* program of HMMER software. Next, the fast multiple sequence alignment software MAFFT (v7.310) [66] set to its default parameters was used to complete the multiple sequence alignment of the putative m^6^A regulators in five plant species (*Physcomitrella patens*, *Zea mays*, *Arabidopsis thaliana*, *Solanum lycopersicum*, and *Lycium barbarum*). FastTree (v2.1.10) [30] set to its default parameters was used to construct approximate maximum likelihood phylogenetic tree based on the multiple sequence alignment results (Figure S3).

### Analyses of m^6^A-Seq data

Raw sequencing reads were cleaned using the fastp tool (v0.20.1) [19] to remove any reads containing adapter or low-quality sequences. Cleaned reads were then mapped onto the reference genome (accession number PRJNA640228) using HISAT2 (v2.2.1) [20, 21] under its default settings. To identify the m^6^A peak, the R package ‘exomePeak’—with a major update released (exomePeak2)—was used with default parameters [67]. The *DREME* (Discriminative Regular Expression Motif Elicitation) tool in MEME suite (http://meme-suite.org/tools/dreme) [32] was used to distinguish the relatively short (up to 8 bp), ungapped motifs, according the following parameters: minimum length of the motif = 5; maximum length of the motif = 7; *E*-value threshold = 1E-5. For a specified motif (e.g., RRACH or URUAY), to calculate the significance level of its relative enrichment, the *AME* tool in MEME suite (http://meme-suite.org/tools/ame) was used. The discovered m^6^A peaks were divided into five categories based on their positions: 5′-untranslated region (5’UTR), near start codon, coding sequence (CDS), near stop codon, and 3′-untranslated region (3’UTR). The coordinates of these genomic elements were extracted, then bedtools *intersect* (v2.30.0) [60] searched for overlapping between m^6^A peaks and each element. The R package ‘RADAR’ [34] was implemented to reveal the differentially methylated genes from the two wolfberry lines, using the criteria of *p*-value < 0.05 and an absolute fold-change > 2.

### Quantitative analysis of mRNA m^6^A by LC–MS/MS

The LC–MS/MS technique was used to detect global m^6^A levels in wolfberry. For each sample, the RNA sample were digested into single nucleosides in a digestion buffer which contains phosphodiesterase I (0.01 U), nuclease S1 (180 U), 1 mM zinc sulfate, 280 mM sodium chloride and 30 mM sodium acetate. The digestion buffer was placed at 37℃ for 4 h at PH 6.8, and the bacterial alkaline phosphatase (30 U) was used to dephosphorylated for 2 h at 37℃. Enzymes were removed by filtration (Amicon Ultra 10 K MWCO). Then, the nucleosides samples were subjected to liquid chromatography coupled with tandem mass spectrometry (LC–MS/MS) which analysis on a QTRAP 4500 mass spectrometer (SCIEX, Framingham, MA, USA). The quantification of nucleosides was performed using the nucleoside-to-base ion mass transitions of 268.1 to 136.1 for A, 245.1 to 113.0 for U, 244.1 to 112.1 for C, 184.1 to 152.1 for G, 282.1–150.1 for RNA m^6^A. We determined the concentration of m^6^A and A by comparing with the standard curve obtained from their nucleoside standards, and analyzed the ratio of m^6^A to A based on the calculated concentrations. Three independent biological replicates were performed for this experiment.

### Gene ontology enrichment analysis

Gene Ontology (GO) enrichment analysis was performed by implementing the R package ‘clusterProfiler’ [68].

### Conservation analysis

Multiple sequence alignments of protein sequences were performed using MAFFT (v7.310) [65] with default parameters. FastTree (v2.1.10) [30] was used to construct approximate maximum likelihood phylogenetic tree with default parameters. Motif analysis was performed using MEME suite (http://meme-suite.org/tools/dreme) [32]. The conservation analysis results were displayed using TBtools (v1.120) [68].

### Statistical analysis

The Student’s t test and Wilcoxon test were applied to the data, as respectively needed, using the R package ‘ggsignif’ (https://github.com/const-ae/ggsignif).

### Supplementary Information

**Additional file 1: Figure S1.** Flowers of two wolfberry lines under study. (A) Flower of *LB*1. (B) Flower of *LB*5. **Figure S2.** Bioinformatics pipeline for the identification of putative m^6^A regulators in wolfberry. **Figure S3.** Phylogenetic analysis of m^6^A regulators. (A) Phylogenetic tree of ALKBH10B genes. (B) Phylogenetic tree of MT-A70 genes. (C) Phylogenetic tree of FIP37 genes. (D) Phylogenetic tree of HAKAI genes. (E) Phylogenetic tree of VIR genes. Color-marked genes are m^6^A regulators identified in wolfberry. **Figure S4.** Expression levels of 22 m^6^A regulators with TPM greater than 10 at three developmental stages. **Figure S5.** Expression levels of the gene *XLOC_016741* in *LB*1 and *LB*5 at three developmental stages. **Figure S6.** LC-MS/MS assay showing the amount of mRNA m^6^A in *LB*1 and *LB*5. Data are presented as mean ± standard deviation (*n* = 3). **Figure S7.** Comparison of gene length between the m^6^A and non-m^6^A genes of two wolfberry lines. In each boxplot, the horizontal line indicates the median. Statistical analysis was conducted using the Student’s t test; *** *p* < 0.001. **Figure S8.** Comparison of expression levels between the m^6^A and non-m^6^A genes in wolfberry. (A) *LB*1. (B) *LB*5. Statistical analysis was conducted using the Student’s t test; *** *p* < 0.001. **Figure S9.** Gene expression levels based on the distributions of differential m^6^A peaks in wolfberry. (A) *LB*1. (B) *LB*5. Statistical analysis was conducted using the Wilcoxon test; ** *p* < 0.05, *** *p* < 0.001. **Figure S10.** Detection of the canonical m^6^A motif RRACH within the m^6^A peak regions. (A) *LB*1. (B) *LB*5. **Figure S11.** Conservation analysis of two genes between *Arabidopsis*, rice, maize, tomato, and wolfberry. (A) The gene encoding a lipoxygenase. (B) The gene encoding a bHLH transcription factor.

**Additional file 2: Table S1.** Expression patterns of assembled genes in *LB*1 and *LB*5.

**Additional file 3: Table S2.** De novo prediction of encoding capacity of assembled transcripts.

**Additional file 4: Table S3.** Function annotation of assembled transcripts in wolfberry.

**Additional file 5: Table S4.** Transcription factor annotation in wolfberry.

**Additional file 6: Table S5.** Differential expression analysis of two wolfberry lines at three stages.

**Additional file 7: Table S6.** m^6^A peak regions of assembled transcripts.

**Additional file 8: Table S7.** Differential methylation analysis of *LB*1 and *LB*5 at S stage.

**Additional file 9: Table S8.** GO enrichment results of hypermethylated genes.

**Additional file 10: Table S9.** GO enrichment results of hypomethylated genes.

**Additional file 11: Table S10.** List of wolfberry genes with differential expression and differential methylation patterns at the S stage.

## Data Availability

All sequencing data have been deposited into the National Genomics Data Center (NGDC)’s Genome Sequence Archive database under the accession numbers PRJCA015572 (https://ngdc.cncb.ac.cn/search/?dbId=&q=PRJCA015572). The assembled wolfberry transcriptome and corresponding functional annotations are deposited at https://github.com/cma2015/Wolfberry_m6A. The frozen plant materials are available from corresponding authors upon reasonable request.

## Abbreviations

m^6^A: *N*^6^-Methyladenosine
RNA-Seq: RNA-sequencing
m^6^A-Seq: M^6^A-sequencing
*LB*1: Ningqi No.1
*LB*5: Ningqi No.5
T: Tetrad stage
S: Single nucleus pollen stage
M: Mature pollen stage
FC: Fold-change
GO: Gene ontology
bHLH: Basic Helix-Loop-Helix
ERF: Ethylene-Responsive Factor
