# Supplementary material for: Changes in m6A RNA methylation are associated with male sterility in wolfberry
Source: BMC Plant Biol. 2023 Sep 29;23:456. doi: 10.1186/s12870-023-04458-7 (PMC10540408; doi:10.1186/s12870-023-04458-7)
Supplement: Supplementary file 1 — Additional file 1: Figure S1. Flowers of two wolfberry lines under study. (A) Flower of LB1. (B) Flower of LB5. Figure S2. Bioinformatics pipeline for the identification of putative m6A regulators in wolfberry. Figure S3. Phylogenetic analysis of m6A regulators. (A) Phylogenetic tree of ALKBH10B genes. (B) Phylogenetic tree of MT-A70 genes. (C) Phylogenetic tree of FIP37 genes. (D) Phylogenetic tree of HAKAI genes. (E) Phylogenetic tree of VIR genes. Color-marked genes are m6A regulators identified in wolfberry. Figure S4. Expression levels of 22 m6A regulators with TPM greater than 10 at three developmental stages. Figure S5. Expression levels of the gene XLOC_016741 in LB1 and LB5 at three developmental stages. Figure S6. LC-MS/MS assay showing the amount of mRNA m6A in LB1 and LB5. Data are presented as mean ± standard deviation (n = 3). Figure S7. Comparison of gene length between the m6A and non-m6A genes of two wolfberry lines. In each boxplot, the horizontal line indicates the median. Statistical analysis was conducted using the Student’s t test; *** p < 0.001. Figure S8. Comparison of expression levels between the m6A and non-m6A genes in wolfberry. (A) LB1. (B) LB5. Statistical analysis was conducted using the Student’s t test; *** p < 0.001. Figure S9. Gene expression levels based on the distributions of differential m6A peaks in wolfberry. (A) LB1. (B) LB5. Statistical analysis was conducted using the Wilcoxon test; ** p < 0.05, *** p < 0.001. Figure S10. Detection of the canonical m6A motif RRACH within the m6A peak regions. (A) LB1. (B) LB5. Figure S11. Conservation analysis of two genes between Arabidopsis, rice, maize, tomato, and wolfberry. (A) The gene encoding a lipoxygenase. (B) The gene encoding a bHLH transcription factor. [file 12870_2023_4458_MOESM1_ESM.pdf]

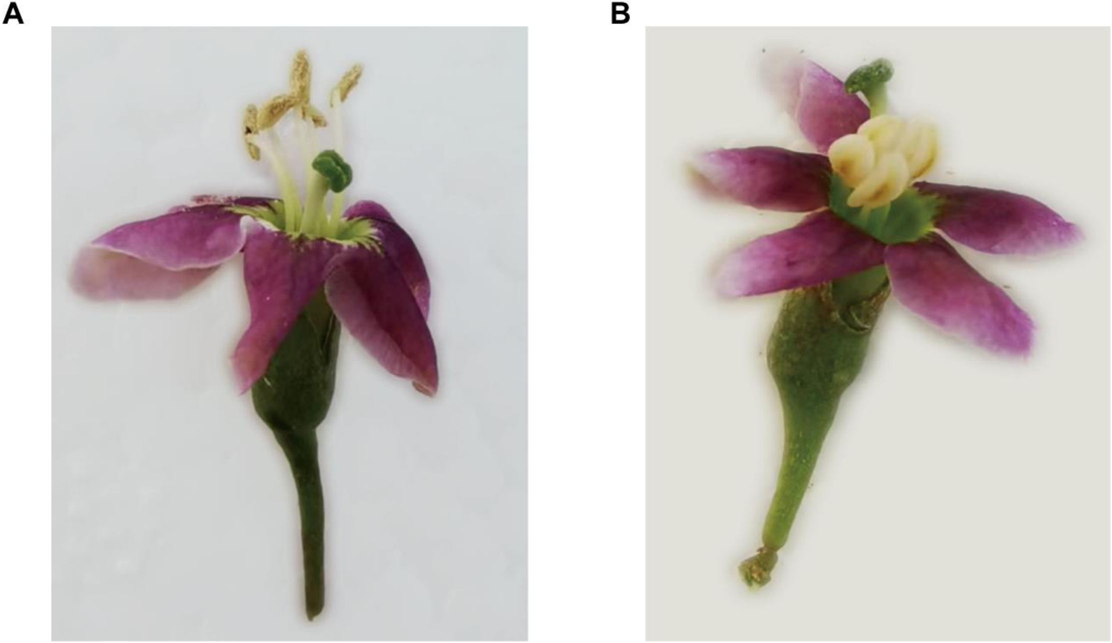

Figure S1. Flowers of two wolfberry lines under study. (A) Flower of *LB*1. (B) Flower of *LB*5

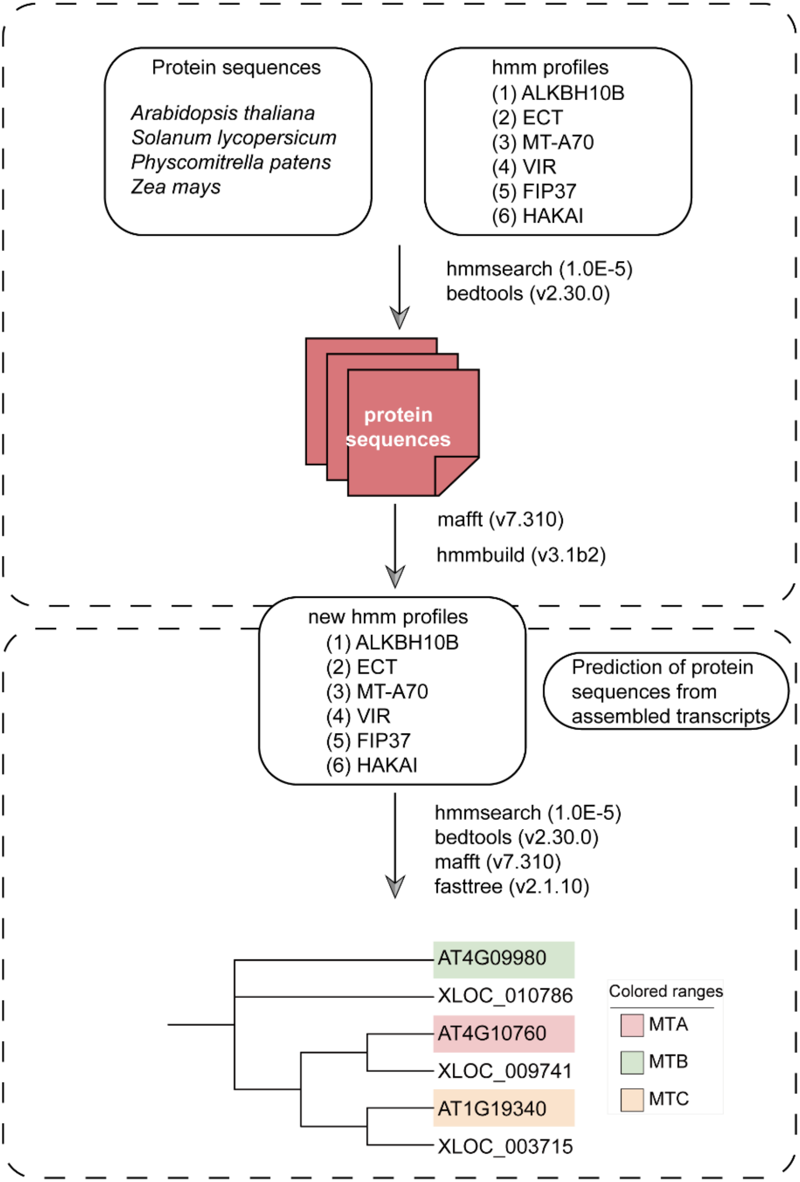

Figure S2. Bioinformatics pipeline for the identification of putative m^{6}A regulators in wolfberry.

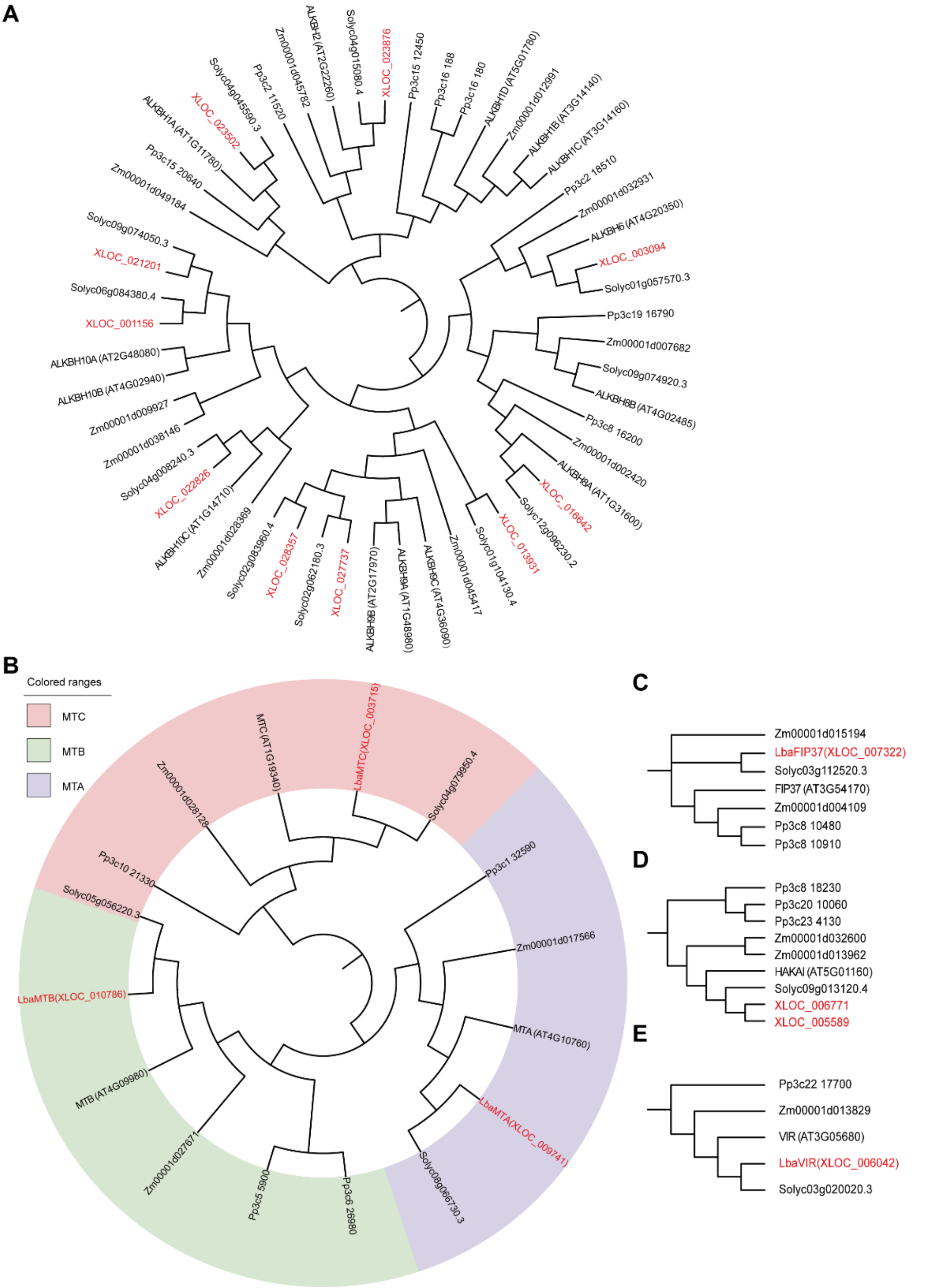

Figure S3. Phylogenetic analysis of m^{6}A regulators. (A) Phylogenetic tree of ALKBH10B genes. (B) Phylogenetic tree of MT-A70 genes. (C) Phylogenetic tree of FIP37 genes. (D) Phylogenetic tree of HAKAI genes. (E) Phylogenetic tree of VIR genes. Color-marked genes are m^{6}A regulators identified in wolfberry.

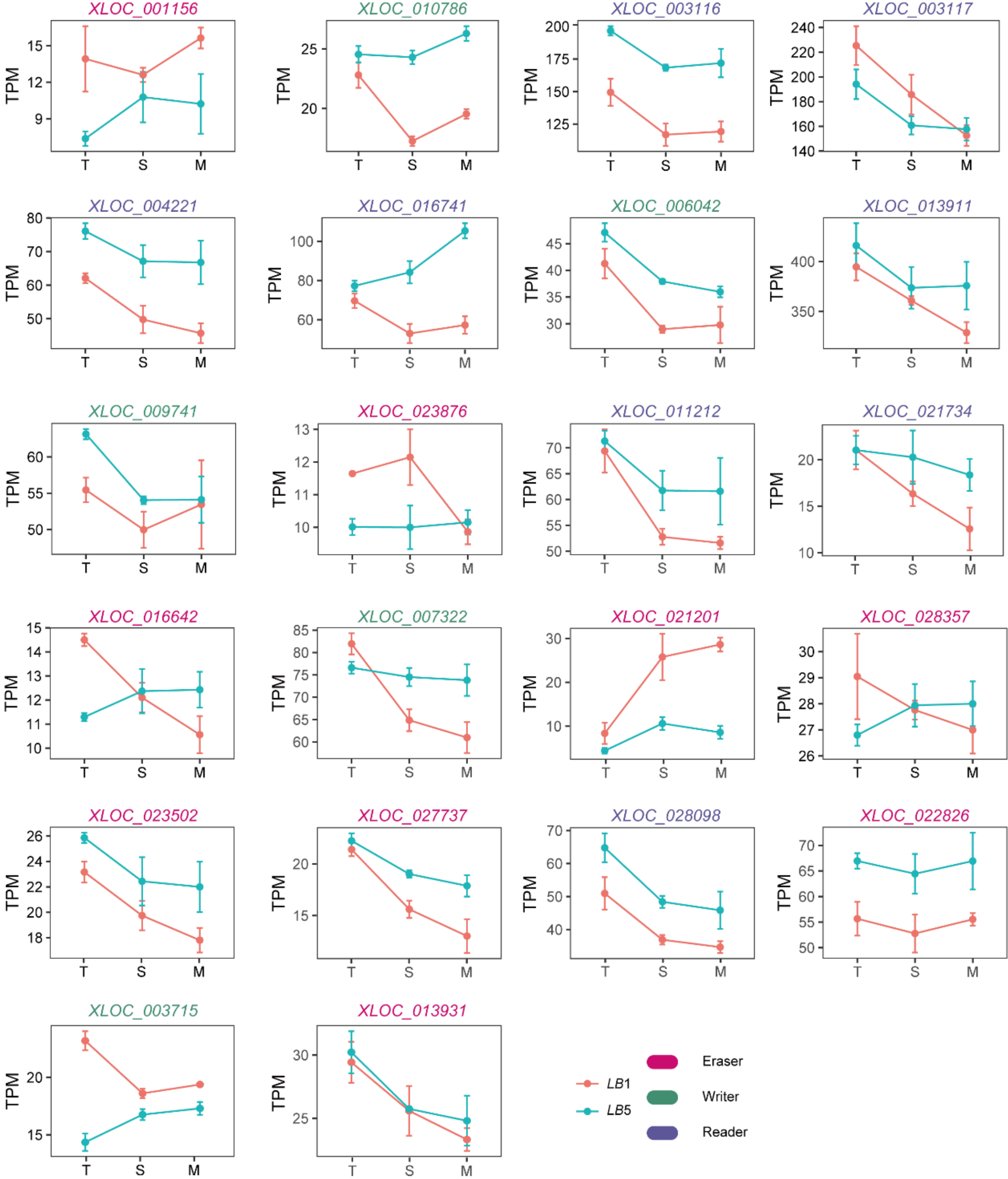

Figure S4. Expression levels of 22 m^6A regulators with TPM greater than 10 at three developmental stages.

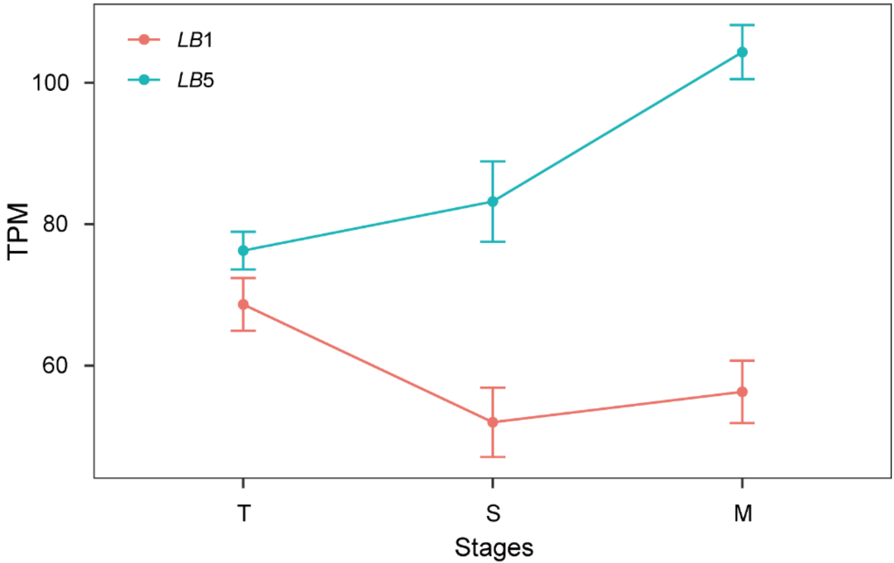

Figure S5. Expression levels of the gene *XLOC_016741* in *LB*1 and *LB*5 at three developmental stages.

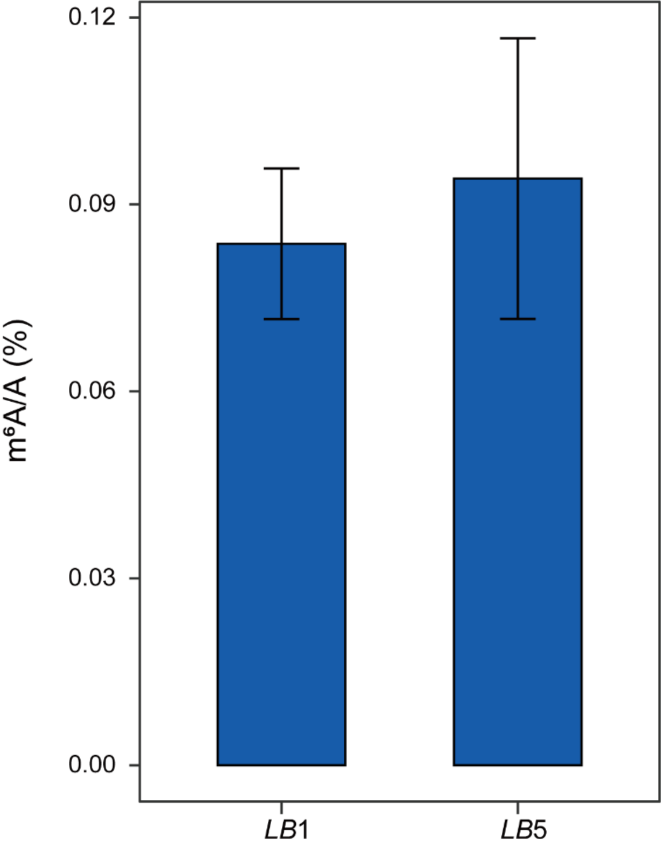

Figure S6. LC-MS/MS assay showing the amount of mRNA m^6A in *LB*1 and *LB*5. Data are presented as mean ± standard deviation (n = 3).

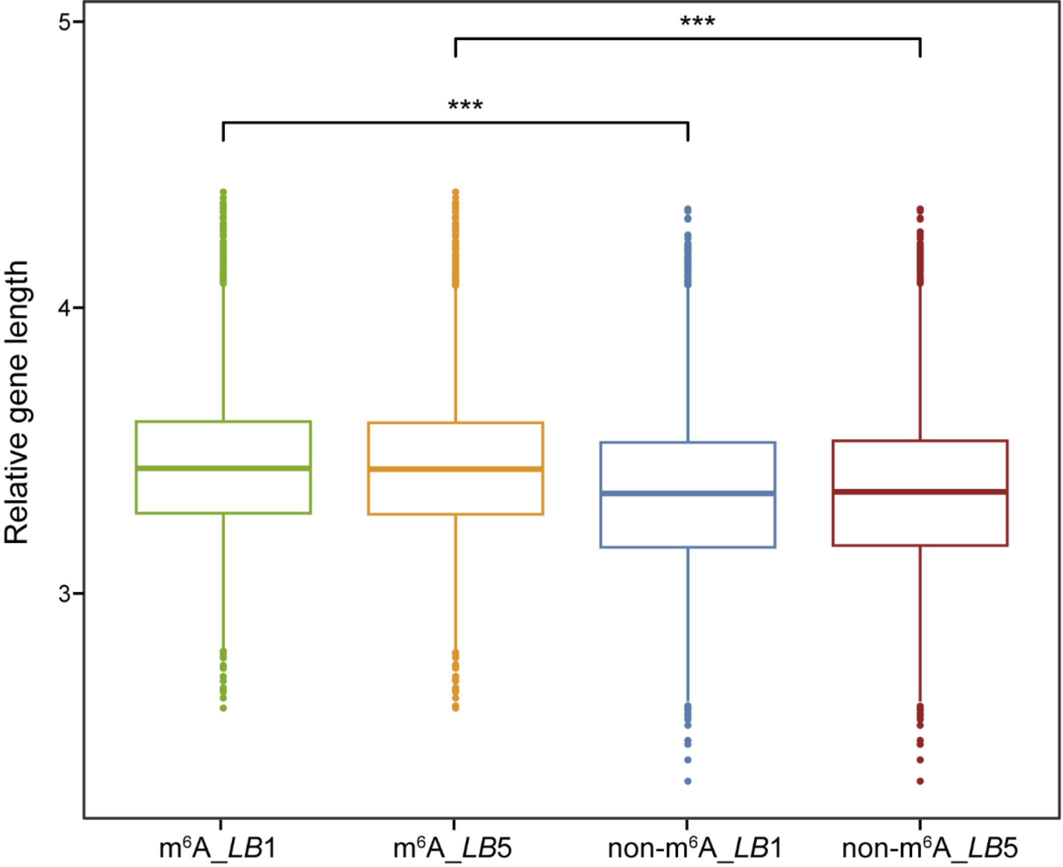

Figure S7. Comparison of gene length between the m^{6}A and non-m^{6}A genes of two wolfberry lines. In each boxplot, the horizontal line indicates the median. Statistical analysis was conducted using the Student's t test; *** $p < 0.001$.

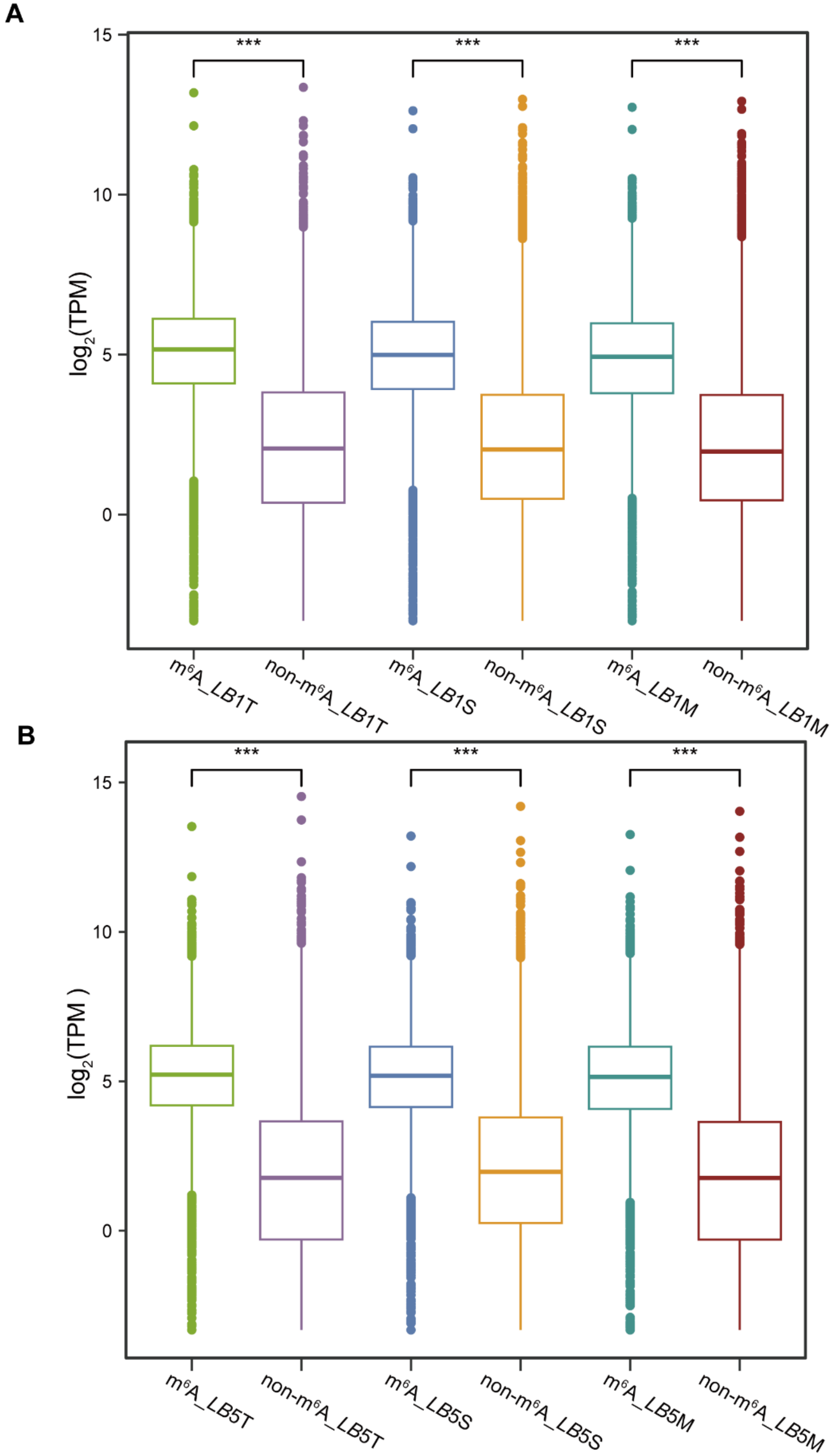

Figure S8. Comparison of expression levels between the m^6A and non-m^6A genes in wolfberry. (A) *LB*1. (B) *LB*5. Statistical analysis was conducted using the Student's t test; *** $p < 0.001$.

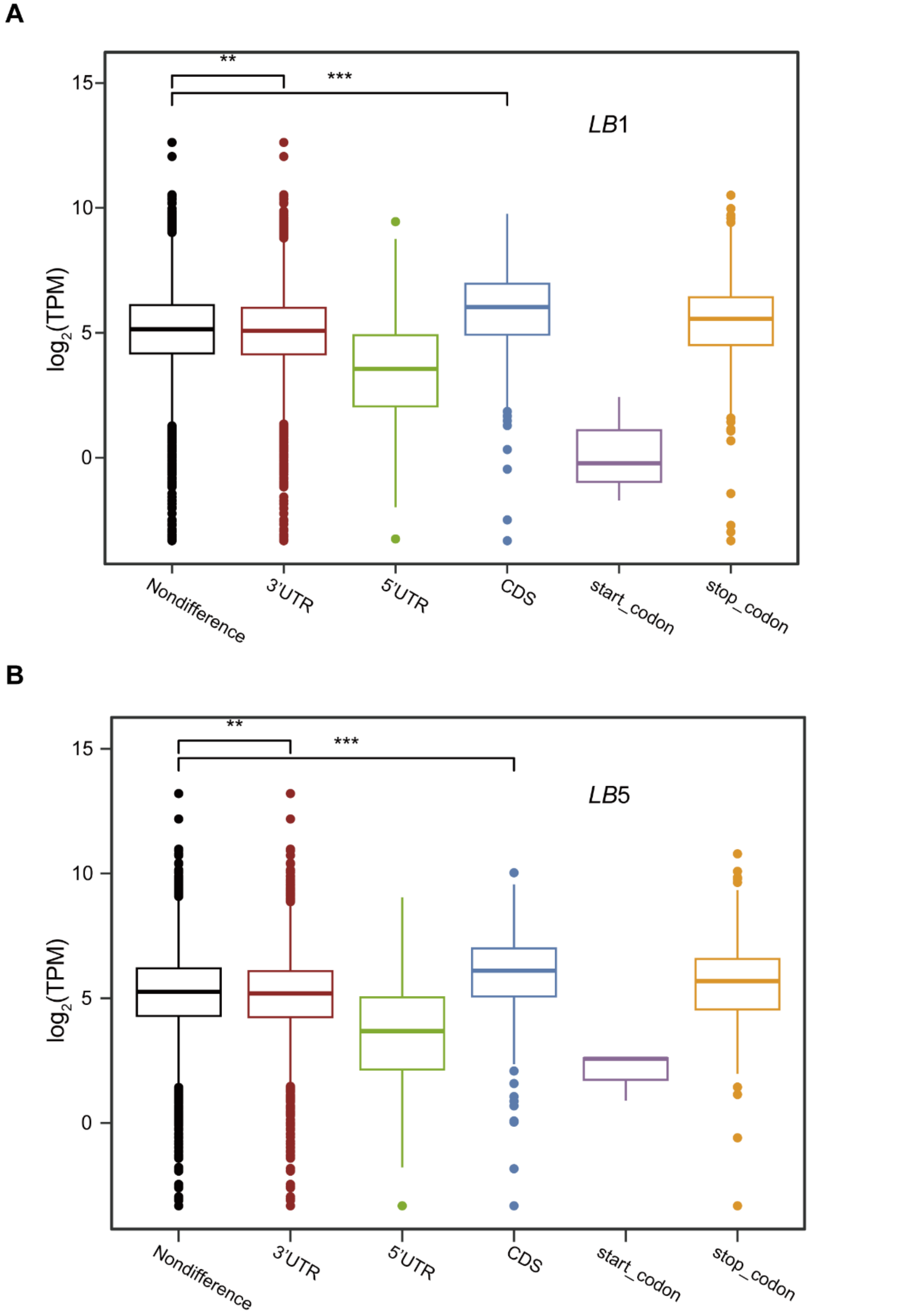

Figure S9. Gene expression levels based on the distributions of differential m^{6}A peaks in wolfberry. (A) *LB*1. (B) *LB*5. Statistical analysis was conducted using the Wilcoxon test; ** $p < 0.05$, *** $p < 0.001$.

A

Rank	Motif	Name	P-value	log P-pvalue	q-value (Benjamini)	# Target Sequences with Motif	% of Targets Sequences with Motif	# Background Sequences with Motif	% of Background Sequences with Motif
1	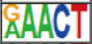	RAACT	1e-28	-6.650e+01	0.0000	7719.0	74.30%	5939.8	66.91%
2	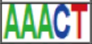	AAACT	1e-25	-5.948e+01	0.0000	6412.0	61.72%	4807.1	54.15%
3	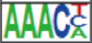	AAACH	1e-20	-4.688e+01	0.0000	9108.0	87.67%	7358.4	82.88%
4	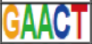	GAACT	1e-18	-4.237e+01	0.0000	4523.0	43.54%	3306.7	37.25%
5	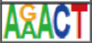	ARACT	1e-13	-3.155e+01	0.0000	7415.0	71.37%	5886.4	66.30%
6	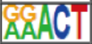	RRACT	1e-12	-2.960e+01	0.0000	8576.0	82.55%	6956.9	78.36%
7	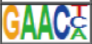	GAACH	1e-10	-2.533e+01	0.0000	7477.0	71.97%	5993.2	67.51%
8	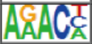	ARACH	1e-9	-2.298e+01	0.0000	9624.0	92.64%	7994.0	90.04%
9	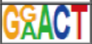	GRACT	1e-9	-2.161e+01	0.0000	5568.0	53.60%	4363.1	49.15%
10	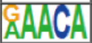	RAACA	1e-9	-2.112e+01	0.0000	8202.0	78.95%	6681.6	75.26%
11	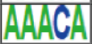	AAACA	1e-9	-2.079e+01	0.0000	7063.0	67.99%	5669.1	63.86%
12	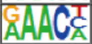	RAACH	1e-8	-1.934e+01	0.0000	9668.0	93.06%	8059.7	90.78%
13	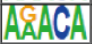	ARACA	1e-7	-1.813e+01	0.0000	8079.0	77.76%	6598.4	74.32%
14	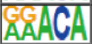	RRACA	1e-6	-1.495e+01	0.0000	9034.0	86.96%	7495.0	84.42%
15	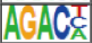	AGACH	1e-5	-1.305e+01	0.0000	6709.0	64.58%	5447.1	61.36%
16	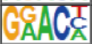	GRACH	1e-5	-1.235e+01	0.0000	8444.0	81.28%	6986.9	78.70%
17	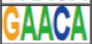	GAACA	1e-5	-1.201e+01	0.0000	4770.0	45.91%	3796.1	42.76%
18	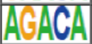	AGACA	1e-5	-1.155e+01	0.0000	4337.0	41.75%	3436.4	38.71%
19	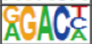	RGACH	1e-4	-1.085e+01	0.0000	8075.0	77.73%	6675.3	75.19%
20	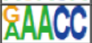	RAACC	1e-3	-8.964e+00	0.0002	5550.0	53.42%	4507.4	50.77%
21	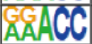	RRACC	1e-3	-8.049e+00	0.0005	6684.0	64.34%	5499.7	61.95%
22	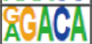	RGACA	1e-3	-7.319e+00	0.0011	5701.0	54.88%	4665.9	52.56%
23	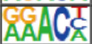	RRACH	1e-3	-6.935e+00	0.0015	9967.0	95.94%	8433.9	95.00%
24	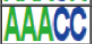	AAACC	1e-2	-6.542e+00	0.0022	4226.0	40.68%	3423.7	38.56%
25	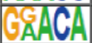	GRACA	1e-2	-6.397e+00	0.0024	6009.0	57.84%	4947.4	55.73%
26	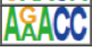	ARACC	1e-2	-5.645e+00	0.0049	5272.0	50.75%	4331.5	48.79%

B

Rank	Motif	Name	P-value	log P-pvalue	q-value (Benjamini)	# Target Sequences with Motif	% of Targets Sequences with Motif	# Background Sequences with Motif	% of Background Sequences with Motif
1	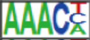	AAACH	1e-20	-4.818e+01	0.0000	8191.0	88.07%	6508.5	82.96%
2	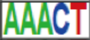	AAACT	1e-19	-4.488e+01	0.0000	5778.0	62.12%	4332.5	55.23%
3	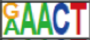	RAACT	1e-16	-3.803e+01	0.0000	6964.0	74.87%	5422.1	69.11%
4	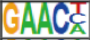	GAACH	1e-16	-3.768e+01	0.0000	6786.0	72.96%	5265.2	67.11%
5	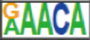	RAACA	1e-15	-3.608e+01	0.0000	7373.0	79.27%	5805.2	74.00%
6	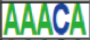	AAACA	1e-13	-3.092e+01	0.0000	6350.0	68.27%	4928.8	62.83%
7	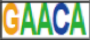	GAACA	1e-12	-2.923e+01	0.0000	4309.0	46.33%	3201.9	40.81%
8	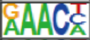	RAACH	1e-12	-2.803e+01	0.0000	8711.0	93.66%	7119.5	90.75%
9	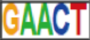	GAACT	1e-11	-2.661e+01	0.0000	4065.0	43.70%	3020.2	38.50%
10	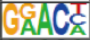	GRACH	1e-10	-2.453e+01	0.0000	7668.0	82.44%	6154.0	78.44%
11	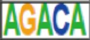	AGACA	1e-10	-2.304e+01	0.0000	3908.0	42.02%	2921.6	37.24%
12	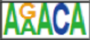	ARACA	1e-9	-2.293e+01	0.0000	7269.0	78.15%	5805.1	74.00%
13	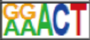	RRACT	1e-8	-1.997e+01	0.0000	7712.0	82.92%	6228.5	79.39%
14	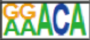	RRACA	1e-8	-1.942e+01	0.0000	8139.0	87.51%	6623.6	84.43%
15	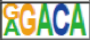	RGACA	1e-7	-1.719e+01	0.0000	5152.0	55.39%	4021.9	51.27%
16	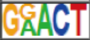	GRACT	1e-7	-1.702e+01	0.0000	5007.0	53.83%	3900.9	49.72%
17	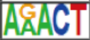	ARACT	1e-6	-1.601e+01	0.0000	6650.0	71.50%	5322.4	67.84%
18	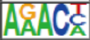	ARACH	1e-6	-1.421e+01	0.0000	8627.0	92.75%	7116.4	90.71%
19	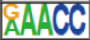	RAACC	1e-6	-1.392e+01	0.0000	5023.0	54.00%	3949.3	50.34%
20	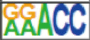	RRACC	1e-5	-1.252e+01	0.0000	6066.0	65.22%	4856.9	61.91%
21	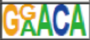	GRACA	1e-4	-1.147e+01	0.0000	5421.0	58.28%	4318.8	55.05%
22	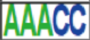	AAACC	1e-4	-9.342e+00	0.0001	3810.0	40.96%	2992.5	38.14%
23	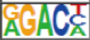	RGACH	1e-4	-9.216e+00	0.0002	7260.0	78.06%	5934.9	75.65%
24	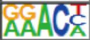	RRACH	1e-3	-8.768e+00	0.0002	8949.0	96.22%	7459.1	95.08%
25	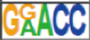	GRACC	1e-2	-5.738e+00	0.0046	3358.0	36.10%	2675.9	34.11%
26	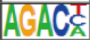	AGACH	1e-2	-5.347e+00	0.0066	6019.0	64.71%	4926.0	62.79%
27	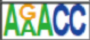	ARACC	1e-2	-4.998e+00	0.0090	4737.0	50.93%	3846.9	49.03%
28	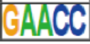	GAACC	1e-2	-4.964e+00	0.0090	2517.0	27.06%	1992.1	25.39%

Figure S10. Detection of the canonical m^{6}A motif RRACH within the m^{6}A peak regions. (A) *LB*1. (B) *LB*5.

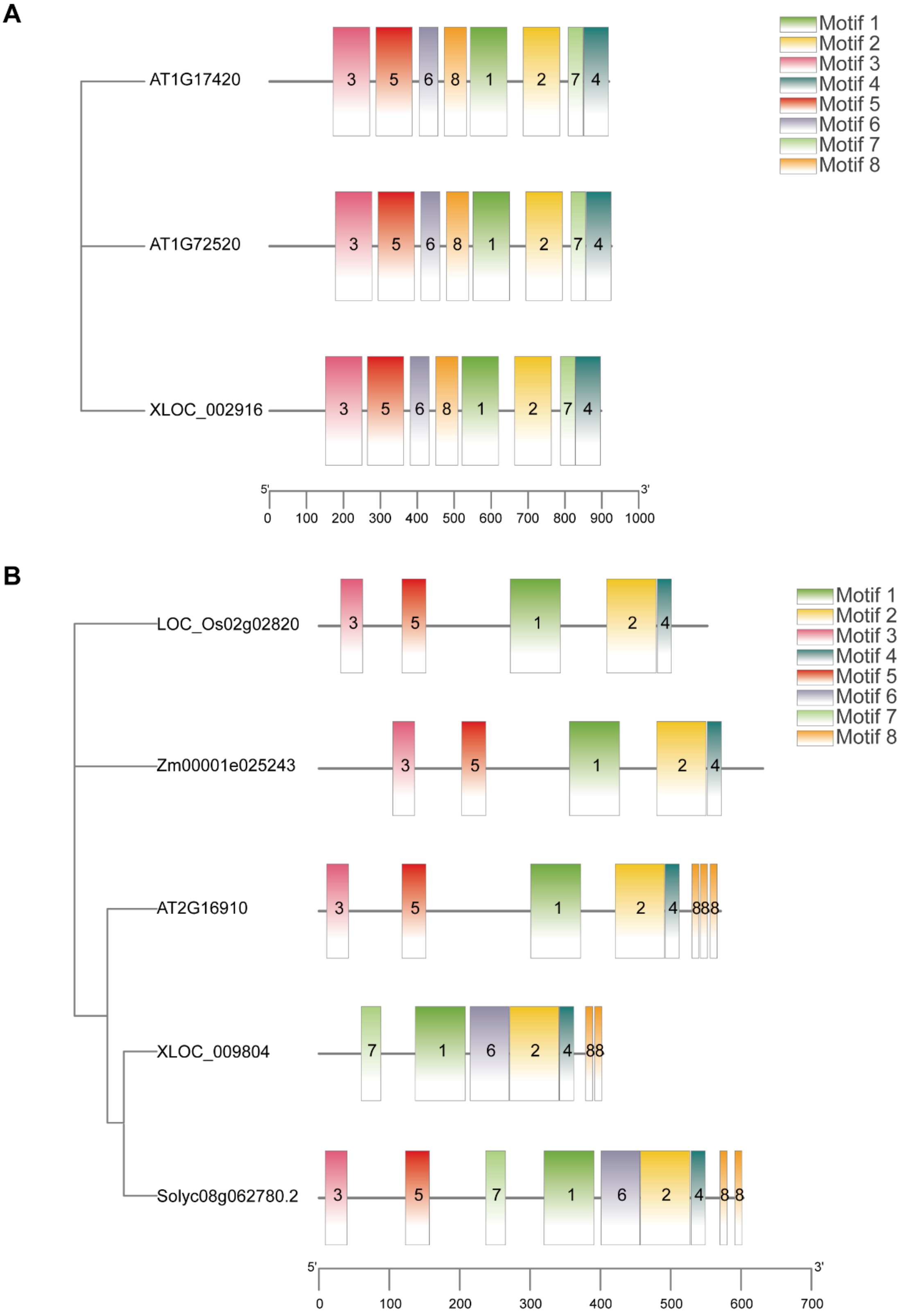

Figure S11. Conservation analysis of two genes between *Arabidopsis*, rice, maize, tomato, and wolfberry. (A) The gene encoding a lipoxygenase. (B) The gene encoding a bHLH transcription factor.
